# Antagonism of Two Plant-Growth Promoting *Bacillus velezensis* Isolates Against *Ralstonia solanacearum* and *Fusarium oxysporum*

**DOI:** 10.1038/s41598-018-22782-z

**Published:** 2018-03-12

**Authors:** Yu Cao, Hualiang Pi, Pete Chandrangsu, Yongtao Li, Yuqi Wang, Han Zhou, Hanqin Xiong, John D. Helmann, Yanfei Cai

**Affiliations:** 1 College of Natural Resources and Environment, South China Agricultural University, Guangzhou, 510642 PR China; 2 Department of Microbiology, Cornell University, Ithaca, NY 14853-8101 USA; 3 College of Environmental Science and Engineering, Guangzhou University, Guangzhou, 510006 PR China; 4 Guangzhou Daodong New Energy Co. Ltd, Guangzhou, 510670 PR China

## Abstract

Plant growth promoting rhizobacteria (PGPR) provide an effective and environmentally sustainable method to protect crops against pathogens. The spore-forming *Bacilli* are attractive PGPR due to their ease of storage and application. Here, we characterized two rhizosphere-associated *Bacillus velezensis* isolates (Y6 and F7) that possess strong antagonistic activity against *Ralstonia solanacearum* and *Fusarium oxysporum* under both laboratory and greenhouse conditions. We identified three lipopeptide (LP) compounds (surfactin, iturin and fengycin) as responsible for the antimicrobial activity of these two strains. We further dissected the contribution of LPs to various biological processes important for rhizosphere colonization. Although either iturin or fengycin is sufficient for antibacterial activity, cell motility and biofilm formation, only iturin plays a primary role in defense against the fungal pathogen *F*. *oxysporum*. Additionally, we found that LP production is significantly stimulated during interaction with *R. solanacearum*. These results demonstrate the different roles of LPs in the biology of *B. velezensis* and highlight the potential of these two isolates as biocontrol agents against phytopathogens.

## Introduction

There is a global need to meet rising food demand from a growing human population in the face of rising energy costs, pressure on natural resources, and concern over global warming^1^. Pressure to meet increased demand has led to the use of chemical pesticides and fertilizers^2^, and the development of genetically resistant plant cultivars^3,4^. Both strategies have limitations. Agrochemicals are not effective against all diseases and can pose a health and environmental risk as toxic residues can accumulate in the soil and enter the food supply. The manufacture of conventional nitrogen-based fertilizers is dependent on fossil fuel energy resources and can have adverse effects on environmental and human health. Disease resistance of genetically modified plants is often overcome by the pathogen within a few years and there is a general lack of public acceptance for genetically modified plants in the food supply^5^. The application of plant growth promoting rhizobacteria (PGPR) and fungi offers an environmentally sustainable alternative to the use of genetically modified plants or synthetic chemicals^6^.

Gram-positive *Bacillus* species are attractive PGPR^7^. First, they form heat and desiccation resistant endospores that can be formulated as a stable dry white powder with a long shelf life^8^. Second, *Bacillus* species are common inhabitants of the microflora of crops including tomato (*Solanum lycopersicum*), banana (*Musa acuminata*), sweet corn (*Zea mays* convar*. saccharata* var*. rugosa*), grape (*Vitis spp*.), and wheat (*Triticum aestivum*). Thus, PGPR can suppress phytopathogens by outcompeting them for colonization of the rhizosphere. More importantly, PGPR produce a wide range of antibiotic compounds including non-ribosomally encoded lipopeptides (LPs)^9,10^. These LPs are the major contributor to *Bacillus* biocontrol activity. For instance, *B. amyloliquefaciens* FZB42, recently reassigned as *B velezensis*^11^, produces fengycin and bacillomycin D, which show synergistic antagonistic activity against the fungal pathogen *Fusarium oxysporum*^9^. *B. velezensis* SQR9 produces bacillomycin D, which contributes to biocontrol activity against *F. oxysporum*^12^.

*Ralstonia solanacearum* is an economically important bacterial phytopathogen^13^ and infects a broad range of host plants including crops such as potato (*Solanum tuberosum*), eggplant (*Solanum melongena*), and tomato (*Solanum lycopersicum*)^14,15^. Tomato wilt caused by *R. solanacearum* is one of the most damaging tomato diseases, particularly during the hot-humid summer season in subtropical regions such as South China. No completely effective chemical controls are available^16,17^. A few resistant tomato cultivars have been developed such as FL7514 and BHN466^18,19^; however, host resistance is often linked with unwanted traits such as reduced fruit size^20^. Some LP-producing *Bacillus* species have been reported as promising biocontrol agents^21–23^. However, the role of LPs in their biocontrol activity against *R. solanacearum* is unclear.

*F. oxysporum* f. sp. *cubense* 4 (Foc), a soil-borne fungus, is the causative agent of banana *Fusarium* wilt (also called Panama disease). *Fusarium* wilt is a global threat to the banana industry. It has seriously affected banana plantations in many countries and caused severe crop losses^24,25^. In South China, *Fusarium* wilt is the major disease faced by many banana plantations^26^. The disease incidence is in the range of 20 to 40%, with some plantations reaching a rate of 90%^27^. Most commercial fungicides are ineffective at controlling the disease. Currently, the best solution is the use of genetically resistant cultivars, but these plants are not resistant to all races of *F. oxysporum*. Emerging evidence has shown that LP-producing PGPR offers a potential environmentally-friendly biocontrol method against *F. oxysporum*^9,12^. However, PGPR isolated from non-native locations may not perform well, since they may not survive in the local soil environment due to circumstantial variables (e.g. pH, moisture, temperature), may be outcompeted by indigenous bacteria, or may not be effective against local pathogens^28,29^. These considerations motivated us to isolate and characterize PGPR endemic to the area where they will be applied.

Here we isolated and characterized two rhizosphere-associated *B. velezensis* strains (Y6 and F7) from a local farm in South China. These strains exhibit potent biocontrol activity against tomato bacterial wilt and banana *Fusarium* wilt under greenhouse conditions. We identified and quantified the antimicrobial LPs secreted by the two strains and defined their roles in biological processes relevant to rhizosphere colonization and plant protection. Either iturin or fengycin is sufficient for antibacterial activity, cell motility and biofilm formation, whereas iturin but not fengycin plays an essential role in antagonism against Foc. These two potential biocontrol agents offer a promising strategy to combat plant pathogens.

## Results

### Isolation and identification of two *Bacillus* strains with significant antibacterial activity

**Figure 1:**
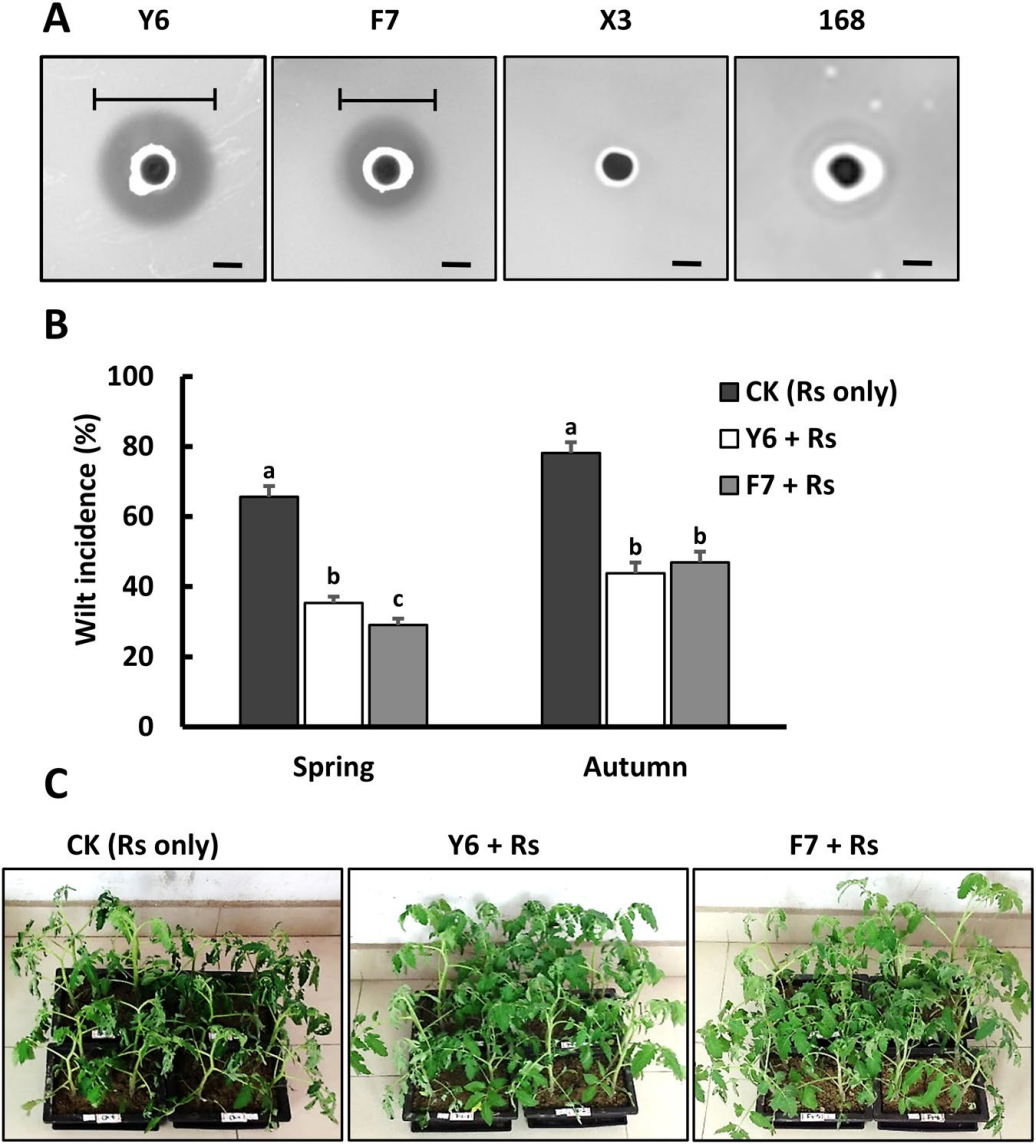
The two *B. velezensis* isolates (Y6 and F7) exhibit strong antibacterial activity against *R. solanacearum in vitro* and *in vivo*. (**A**) To evaluate the inhibitory activity of the two isolates against *R. solanacearum*, a spot-on-lawn assay was performed. The clearance zone indicated by the black lines was measured after 24 h incubation at 30 °C (Y6, 10 ± 0.2 mm, F7, 8 ± 0.2 mm, mean ± SD with n = 3). *B megaterium* X3 and *B. subtilis* 168 served as controls and showed no inhibition activity. The scale bar is 5 mm. (**B**) The biocontrol ability of the two isolates to suppress tomato bacterial wilt was evaluated in pot experiments during spring and autumn using non-sterile local soil (see Additional Methods in the SI materials). Three groups were included: control (CK), only inoculated with *R. solanacearum* (Rs); Y6 + Rs, inoculated with Y6 and Rs; and F7 + Rs, inoculated with F7 and Rs. The wilt incidence was calculated on the 30^th^ day after transplanting as described in Materials and Methods. The data are expressed as the mean ± SD (n = 24). Significant differences between the control and treated group (Y6 or F7) are determined by Tukey’s Studentized Range (HSD) Test as indicated: α = 0.05, n = 24. (**C**) Representative photographs of the tomato plants to show typical wilt symptoms in CK group while only minor-to-negligible symptoms in the two treated groups after 25 days of transplanting in the spring pot experiments.

Many rhizosphere-associated *Bacillus* isolates exhibit antimicrobial activity towards phytopathogens^10,12,30^. To isolate antagonistic *Bacillus* species for use in the subtropical regions of China, we collected rhizosphere soil from healthy tomato plants in a local farm in Guangzhou. Among 60 isolated strains, two exhibited the strongest inhibitory effects against *R. solanacearum* GMI1000 on Casamino acid-Peptone-Glucose (CPG) plates using an optimized spot-on-lawn assay, where a high-density culture of the test strain is spotted on a low-density lawn of the indicator strain (*R. solanacearum*) (Fig. 1A). These two isolates were named Y6 and F7. Phylogenetic trees, constructed based on 16S *rRNA* and *gyrA* gene sequences, indicate that Y6 and F7 cluster with *plantarum* subspecies of *B. velezensis* such as S499, UMAF6614, SQR9 and FZB42 (Fig. S1).

These two isolates showed strong antimicrobial activity against *R. solanacearum in vitro*. Thus, we tested their ability to suppress tomato wilt caused by *R. solanacearum* in pot experiments under greenhouse settings during spring and autumn. After treatment with either Y6 or F7, the wilt incidence was significantly reduced in both sets of experiments (Fig. 1B,C). For example, in pot experiments done in spring, the wilt incidence in the control group (~65%) was reduced to 29% or 35% for Y6- or F7-treated groups, respectively (Fig. 1B,C). These results reveal that the disease control ability (biocontrol efficacy) of these two isolates approaches 50%.

### Identification of the lipopeptide compounds secreted by Y6 and F7

**Figure 2:**
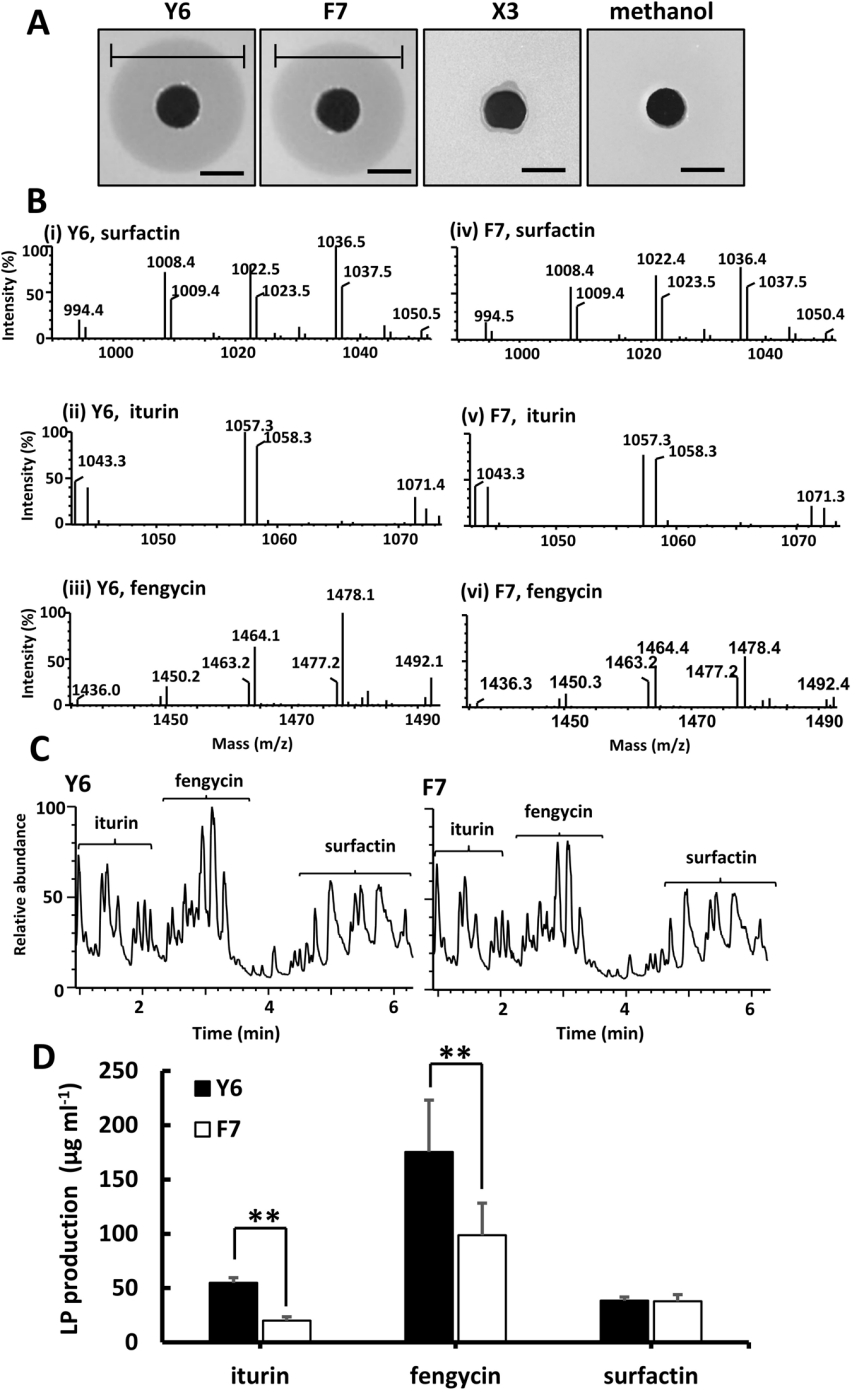
Identification and quantification of LP compounds from the two isolates. (**A**) The methanol extracts from the two isolates showed strong antibacterial activity against *R. solanacearum*, as judged by an agar diffusion assay. Five µl of the methanol extracts of Y6, F7, *B. megaterium* X3 (X3; control), and methanol only (control) were tested. The latter two served as controls and did not show any inhibitory effects. The clearance zone indicated by the black lines is evident when treated with the methanol extract from either isolate (Y6, 14 ± 0.1 mm; F7, 13 ± 0.1 mm; mean ± SD with n = 3). The scale bar is 5 mm. (**B**) Identification of LP compounds from the methanol extract of Y6 or F7 using UPLC-MS analysis. Mass spectrum [M+H]^+^ of LPs are shown, surfactin ((i, Y6) and (iv, F7)), fengycin((ii, Y6) and (v, F7)), and iturin((iii, Y6) and (vi, F7)). (**C**) Representative chromatograms of the LPs (iturin, fengycin and surfactin) in methanol extract (3 μl) from strain Y6 or F7 using UPLC-MS analysis. (**D**) Quantification of the LP compounds (surfactin, iturn and fengycin) secreted by Y6 or F7. Cell were grown in LB medium at 30 °C for 48 h, harvested by centrifugation, and subjected to methanol extraction for LP analysis. The LPs were then quantified based on the standard curves of commercial standards surfactin, iturin and fengycin. Significant differences between Y6 and F7 were determined by two-tailed t-test as indicated: **P < 0.01, n = 3.

In some PGPR, the biocontrol activity is linked to their ability to produce lipopeptide (LP) compounds^21,31^. We isolated the secreted metabolites produced by the Y6 and F7 strains grown in LB medium and both extracts showed very strong activity against *R. solanacearum* with a clearance zone of 13–14 mm in diameter (Fig. 2A). We then identified the LP compounds from the extracts using ultra-performance liquid chromatography coupled with mass spectrometry (UPLC-MS). Three major LPs were detected: iturin, fengycin, and surfactin (Fig. 2B). Multiple isoforms of each LP were observed, including five for surfactin (C12 to C16), three for iturin (C14 to C16), and six for fengycin (C12 to C16) (Fig. 2B and Table S3). Consistent with the observation that Y6 showed higher antimicrobial activity against *R. solanacearum* than F7 (Fig. 1A), Y6 produces significantly higher levels of iturin and fengycin than F7 (Fig. 2C,D and Table S3).

To further evaluate the antimicrobial potency of these isolated LPs, we tested the minimum inhibitory concentration (MIC) of the LP standards and compared them with the extracts from Y6 or F7. Both iturin and fengycin standards showed relatively strong antibacterial activity against *R. solanacearum* with a MIC of 100 µg ml^−1^ (Fig. S2B,C), while surfactin showed moderate activity with a MIC of 500 µg ml^−1^ (Fig. S2A). By contrast, the MIC of the extract from either Y6 or F7 is 75 µg ml^−1^ (Fig. S3D,E), considerably lower than that of any single LP (Fig. S2A–C), suggesting that these three LPs may have synergistic antagonistic activity against pathogenic organisms.

### LP production is strongly stimulated in the presence of *R. solanacearum*

**Figure 3:**
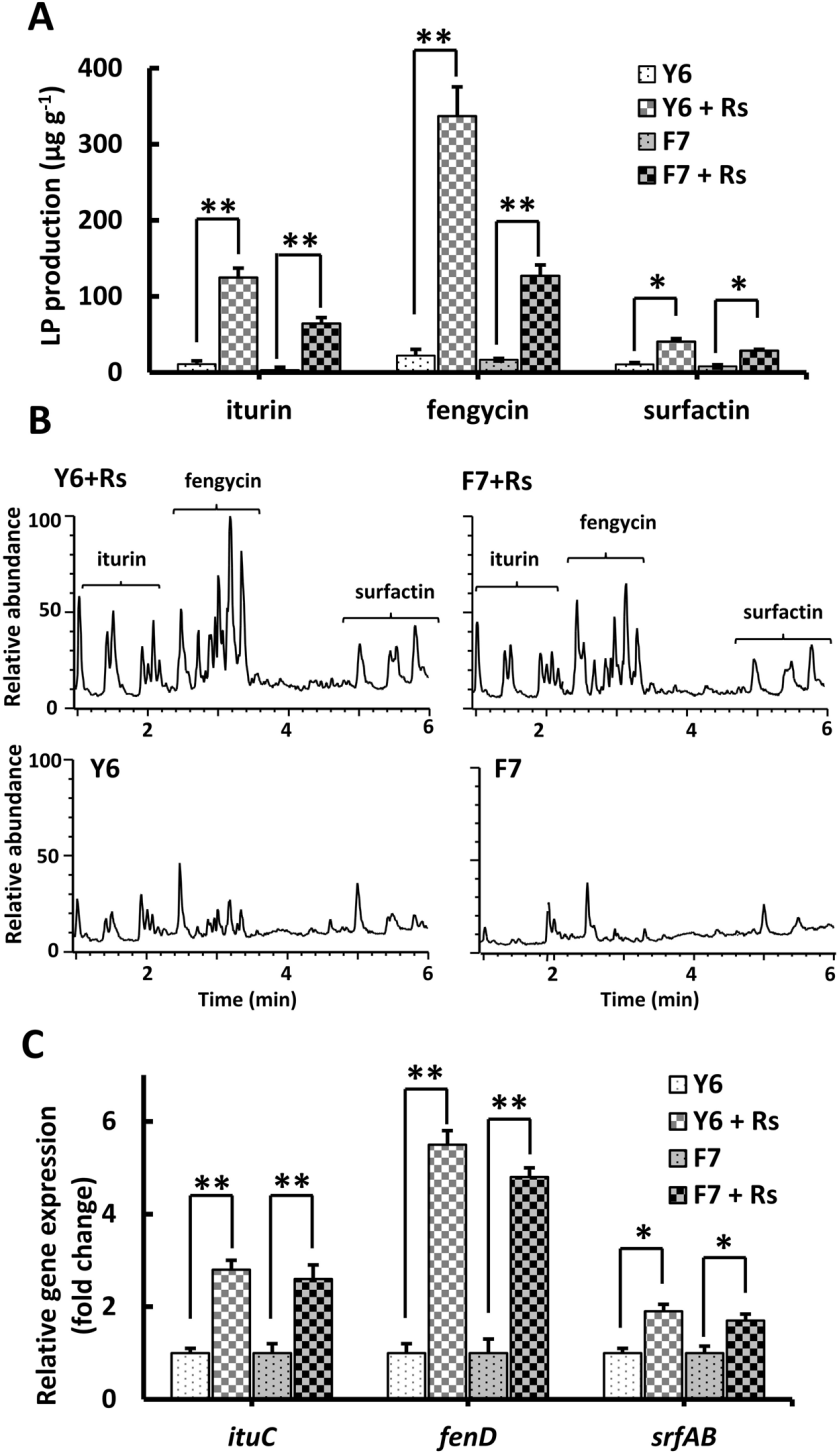
*R. solanacearum* strongly stimulates production of LPs. (**A**) LP compounds were extracted from the inhibition zone (Fig. 1A) and quantified by UPLC-MS analysis as described in Materials and Methods. Briefly, after the inhibition zone against *R. solanacearum* (Rs) became evident after 24 h incubation at 30 °C, a 300 mg of agar sample was harvested from the inhibition zone, mixed with 1 ml of acetonitrile/water (1:1 v/v), and sonicated for 30 s followed by centrifugation and filtration. The supernatant was collected as the acetonitrile/water extract and subjected to UPLS-MS analysis. The control was performed in a similar way but without the pathogen Rs in the top soft agar layer. (**A**) 300 mg of agar sample was collected around the well (2–4 mm). The two isolates produce more LPs, particularly fengycin and iturin, when they encounter the pathogen *R. solanacearum*. B. Representative chromatograms illustrate the differences in LP production by strain Y6 or F7 in the presence or absence of the co-culturing pathogen *R. solanacearum*. (**C**) The presence of *R. solanacearum* affects expression of LP biosynthesis genes. Expression of the LP biosynthesis genes (*srfAB*, *ituC* and *fenD* for synthesis of surfactin, iturin, and fengycin, respectively) was monitored in the two isolates in the presence or absence of the co-culturing pathogen *Rs*. RNA extraction and qPCR were performed as described in Materials and Methods. The relative gene expression (in fold change) was calculated using the 2^−ΔΔCt^ method. For (**A**,**C**), significant differences between the Rs infected and control groups were determined by two-tailed t-test as indicated: **P < 0.01, *P < 0.05, n = 3.

LP production by PGPR can be modulated by the presence of a pathogenic organism^32^. Thus, we evaluated the influence of *R. solanacearum* on the production of LPs by Y6 and F7. CPG agar samples were harvested from the clearance zone (Fig. 1A) and LP compounds were extracted using an acetonitrile/water based method and then subjected to UPLC-MS analysis. Co-culture with *R. solanacearum* significantly stimulated LP production, particularly fengycin and iturin. When encountering the pathogen, iturin production increased 11- and 20-fold (F7 and Y6 respectively) and fengycin production increased 15- and 38-fold (F7 and Y6, respectively) (Fig. 3A,B and Table 1). A moderate increase of ~4-fold was observed for surfactin production by Y6 and F7 (Fig. 3A,B and Table 1).

**Table 1:** LP production is significantly stimulated during interaction with *R. solanacearum* (RS).

LPs	isoforms	Y6 + Rs (µg g^−1^)	Ratio (%)	Y6 (no Rs) (µg g^−1^)	Ratio (%)	F7 + Rs (µg g^−1^)	Ratio (%)	F7 (no Rs) (µg g^−1^)	Ratio (%)
iturin	C14	38.2 ± 6.6	31%	5.1 ± 2.8	46%	19.7 ± 2.1	31%	1.7 ± 2.1	52%
	C15	63.0 ± 5.0	50%	4.9 ± 0.6	44%	35.4 ± 3.5	55%	1.6 ± 1.6	48%
	C16	23.7 ± 0.5	19%	1.2 ± 0.7	11%	9.4 ± 2.1	15%	0.0 ± 0.1	0%
Total iturin		125.0 ± 12.2		11.1 ± 4.1		64.5 ± 7.7		3.3 ± 3.0	
fengycin	C12	5.3 ± 0.5	2%	1.2 ± 1.2	5%	3.5 ± 0.3	3%	1.0 ± 0.9	6%
	C13	9.3 ± 0.1	3%	1.5 ± 0.1	7%	4.4 ± 0.6	3%	2.5 ± 1.2	15%
	C14	54.6 ± 3.3	16%	4.2 ± 1.3	19%	28.7 ± 1.8	23%	5.8 ± 2.8	35%
	C15	117.8 ± 13.8	35%	8.5 ± 2.5	38%	54.9 ± 6.2	43%	1.3 ± 0.4	8%
	C16	58.6 ± 4.4	17%	2.9 ± 2.0	13%	16.0 ± 3.9	13%	2.7 ± 1.5	16%
	C17	91.3 ± 16.5	27%	3.9 ± 1.0	18%	19.5 ± 1.4	15%	3.5 ± 2.0	21%
Total fengycin		336.9 ± 38.6		22.2 ± 8.1		127.0 ± 14.3		16.8 ± 1.6	
surfactin	C12	0.8 ± 0.1	2%	0.3 ± 0.1	10%	0.6 ± 0.0	2%	0.2 ± 0.1	10%
	C13	5.9 ± 1.0	15%	1.1 ± 0.3	35%	3.6 ± 0.6	13%	0.7 ± 0.4	29%
	C13	9.9 ± 1.0	24%	0.4 ± 0.4	13%	7.9 ± 0.5	28%	0.8 ± 0.3	35%
	C15	23.3 ± 1.9	58%	0.5 ± 0.7	17%	16.1 ± 0.6	56%	0.3 ± 1.0	13%
	C16	0.5 ± 0.1	1%	0.8 ± 0.9	25%	0.4 ± 0.0	1%	0.3 ± 0.3	13%
Total surfactin		40.5 ± 4.0		3.1 ± 0.6		28.6 ± 1.8		8.0 ± 2.1	
Total LPs		502 ± 54		36 ± 14		220 ± 24		22 ± 7	

Note: 300 mg of agar sample was harvested from the inhibition zone (Fig. 1A), mixed with 1 ml of acetonitrile/water (1:1 v/v), and sonicated for 30 s followed by centrifugation, filtration, and UPLC-MS analysis. Commercial LP standards were used to generate standard curves for LP quantification. Data are expressed as the mean ± SD (n = 3). Ratio is indicated as the percentage of each individual isoform out of the total LP compound.

Each LP contains multiple isoforms (Fig. 2B, Table 1 and Table S3) that may possess different antimicrobial activity and which may change upon the presence of other bacteria^33,34^. Indeed, upon *R. solanacearum* challenge, the isoform profile of all three LPs changed: the C15 isoform increased in all three LPs and became the dominant iturin and surfactin species (more than 50%) (Table 1). Whether or not the C15 isoform is most effective against *R. solanacearum* is unknown.

The elevated LP production by F6 and Y7 in response to *R. solanacearum* might be due to an increase in expression of LP biosynthesis genes. In fact, the expression of LP genes is highly upregulated in several *Bacillus* isolates during interaction with phytopathogens^35,36^, although LP production was not quantified in these studies. To test this notion, we monitored LP gene expression in the presence of *R. solanacearum*. Indeed, the expression of *fenD* (encoding fengycin synthetase D) in both Y6 and F7 was significantly upregulated (5-6-fold), whereas *ituC* (encoding iturin A synthetase C) and *srfAB* (encoding surfactin synthetase A) expression increased 2-3-fold upon encountering *R. solanacearum* (Fig. 3C). These results are consistent with the LP quantification results (Fig. 3A,B and Table 1) and further confirm that interaction with *R. solanacearum* induces expression of LP genes resulting in stimulated LP production.

### Iturin and fengycin play a redundant role in antagonism against *R. solanacearum*

**Figure 4:**
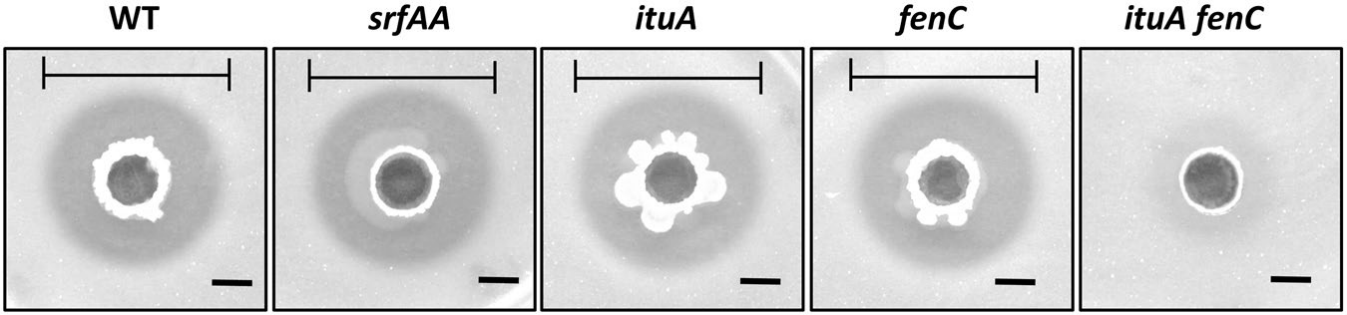
Iturin A and fengycin play a redundant role in defense against *R. solanacearum*. The antagonistic activity of Y6 (WT) and its derived mutants [including *srfAA* (deficient in surfactin synthesis), *ituA* (deficient in iturin synthesis), *fenC* (deficient in fengycin synthesis) and *ituA fenC* (deficient in both iturin and fengycin synthesis)] against the pathogen *R. solanacearum* was tested using a spot-on-lawn assay. The clearance zone indicated by the black lines was evaluated after incubation at 30 °C for 24 h. The experiments were performed at least three times. Representative photographs are shown. The scale bar is 5 mm.

Other compounds in addition to the LPs produced by Y6 and F7 may contribute to antagonistic activity against phytopathogens. Moreover, it is difficult to accurately assess the level of LP production and activity in the soil environment. Therefore, to dissect the contribution of each individual LP compound to antimicrobial activity, we constructed mutants lacking each LP individually or in combination by disrupting LP biosynthesis genes required for iturin, fengycin and surfactin production (*ituA*, *fenC*, and *srfAA*, respectively) in Y6. Y6 was chosen for further study because F7 is naturally resistant to some antibiotics such as spectinomycin, which makes mutant construction more difficult. Loss of any individual LP (*ituA*, *fenC*, or *srfAA*) did not affect the capacity of Y6 to suppress *R. solanacearum* growth on CPG plates, whereas the *ituA fenC* double mutant completely lost the ability to inhibit *R. solanacearum* growth (Fig. 4). These results suggest that iturin and fengycin are the two major antimicrobial compounds and are functionally redundant in suppression of *R. solanacearum*.

Iturin and fengycin have been proposed to have synergistic antagonistic activity against phytopathogens^9^. However, neither *ituA* nor *fenC* single mutants showed reduced antibacterial activity against *R. solanacearum*, suggesting either a lack of synergy in this case or that deficiency in biosynthesis of one LP might be compensated for by increase in production of another LP upon encountering pathogens. Indeed, quantification of LP production in wild-type (Y6), *ituA*, and *fenC* single mutants revealed that iturin production in the *fenC* mutant increased 2-fold (Fig. S3B,C). To test if the crosstalk between LPs occurs at the transcriptional level, we monitored the expression of *ituC* and *fenD* genes in wild-type (Y6), *ituA*, and *fenC* single mutants by qPCR during co-culture with *R. solanacearum*. Expression of *fenD* in the *ituA* mutant was upregulated about 2-fold compared to wild-type, and expression of *ituC* in the *fenC* mutant also increased about 2-fold (Fig. S3A). These results suggest that interactions between the LP biosynthesis pathways at the transcriptional level can allow one LP to compensate, at least in part, for the defective production of another.

### Iturin is the primary factor responsible for antifungal activity against Foc *in vitro* and *in vivo*

**Figure 5:**
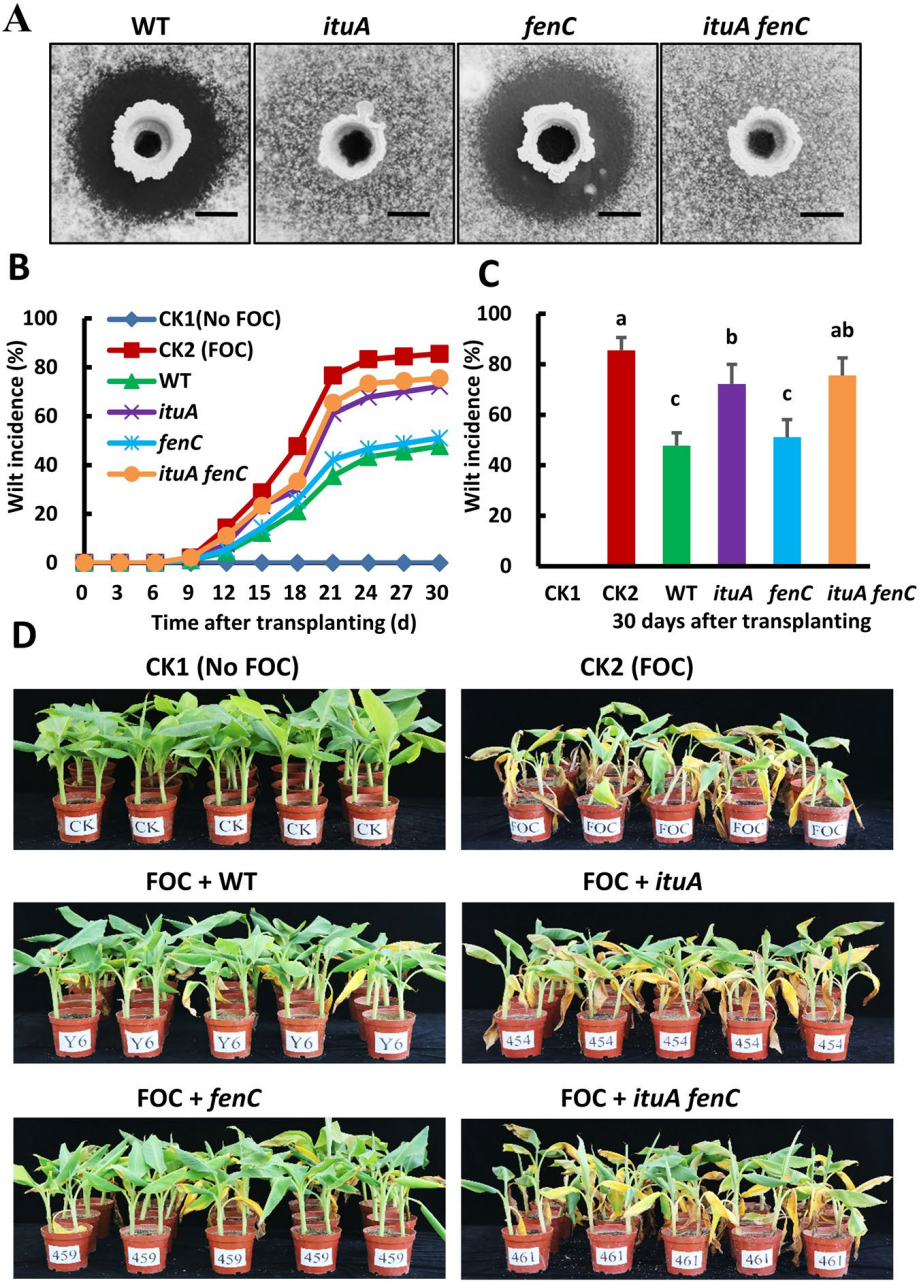
Iturin A is the primary antifungal factor against *F. oxysporum*. (**A**) The antagonistic activity of Y6 (WT) and its derived mutants [including *ituA* (deficient in iturin synthesis), *fenC* (deficient in fengycin synthesis) and *ituA fenC* (deficient in both iturin and fengycin synthesis)] were tested against *F. oxysporum* (Foc) using a spot-on-lawn assay evaluated after incubation at 30 °C for 24 h. The experiments were performed at least three times. Representative photographs are shown. The scale bar is 5 mm. (**B**) To understand the contribution of iturin and fengycin in the biocontrol efficacy against banana *Fusarium* wilt *in vivo*, banana pot experiments were performed under greenhouse condition using non-sterile local soil (see Additional Methods in the SI materials). Same strains listed in panel (A) were tested. Two control groups were included as follows: CK1 (no Foc) and CK2 (Foc). Four treated groups were included: WT (Foc + Y6), *ituA* (Foc + *ituA* single mutant), *fenC* (Foc + *fenC* single mutant) and *ituA fenC* (Foc + *ituA fenC* double mutant). The wilt incidence of the banana plants was monitored every 3 days after transplantation. (**C**) Data presented are the wilt incidence of the banana plants among the same six different groups as described in Fig. 5B on the 30^th^ day after transplanting. The data are expressed as the mean ± SD (n = 30). Significant differences among different groups indicated as different letters on top of the data bars are determined by Tukey’s Studentized Range (HSD) Test (α = 0.05). (**D**) Representative photographs of the banana plants to show the wilt incidence in six different groups including CK1 (No Foc), CK2 (Foc), Foc + WT, Foc + *ituA*, Foc + *fenC* and Foc + *ituA fenC* on the 30^th^ day after transplanting.

PGPR can also provide protection from fungal pathogens^8,32^. To evaluate the antifungal ability of these two isolates, we tested their antagonistic activity against several common fungal plant pathogens using a plate confrontation assay. Both Y6 and F7 significantly inhibited the growth of *Fusarium oxysporum* f.sp*. cubense* (Foc), *F. oxysporum* f.sp. *cucumerinum*, and *Colletotrichum gloeosporioides* on Potato-Dextrose-Agar (PDA) plates (Fig. S4). We then assessed the contribution of iturin and fengycin to antifungal activity using an optimized spot-on-lawn assay (Fig. 5A), which is more sensitive compared to the traditional plate confrontation assay (see Materials and Methods). The *fenC* single mutant did not affect the capacity of Y6 to suppress the wildgrowth of the fungal pathogen Foc (Fig. 5A). Surprisingly, both the *ituA* single mutant and the *ituA fenC* double mutant almost completely lost the ability to inhibit Foc growth, although there is a sensitivity zone with reduced cell growth around the well where the *ituA* single mutant was inoculated (Fig. 5A). Similar results were observed in a plate confrontation test (Fig. S5).

In addition to their fungicidal activity, LPs are known to inhibit fungal growth by inhibiting spore germination. We conducted a spore germination assay using LP commercial standards and Y6 extract. As expected, the Y6 extract showed strong antagonistic activity against Foc and 75 μg ml^−1^ of the LP extract prevented ~60% of the spores from germinating. A similar level of inhibition was observed for the iturin standard (Fig. S6). By contrast, fengycin only showed minor antifungal activity even with the highest concentration tested (200 μg ml^−1^) and surfactin showed no evident inhibitory activity (Fig. S6). These results indicate that iturin is the major LP responsible for antifungal activity *in vitro* and fengycin plays a minor role.

To further investigate the biocontrol activity of Y6 and its derived LP-defective mutants against banana *Fusarium* wilt, we set up banana pot experiments in greenhouse settings. Treatment with WT (Y6) significantly reduced the wilt incidence, 48% by day 30 after transplanting compared to 86% in the untreated group (Fig. 5B–D). Deletion of *fenC* did not significantly affect the wilt incidence (51% by day 30). By contrast, the *ituA* single and *ituA fenC* double mutants only retained minor suppression ability (Fig. 5B–D). The biocontrol efficacy of Y6 was 44% against banana *Fusarium* wilt and iturin alone contributes about 90% of the total biocontrol efficacy. These results suggest that iturin is the major contributor against Foc *in vivo* under controlled-environmental conditions.

### Either iturin or fengycin is sufficient for biofilm formation

**Figure 6:**
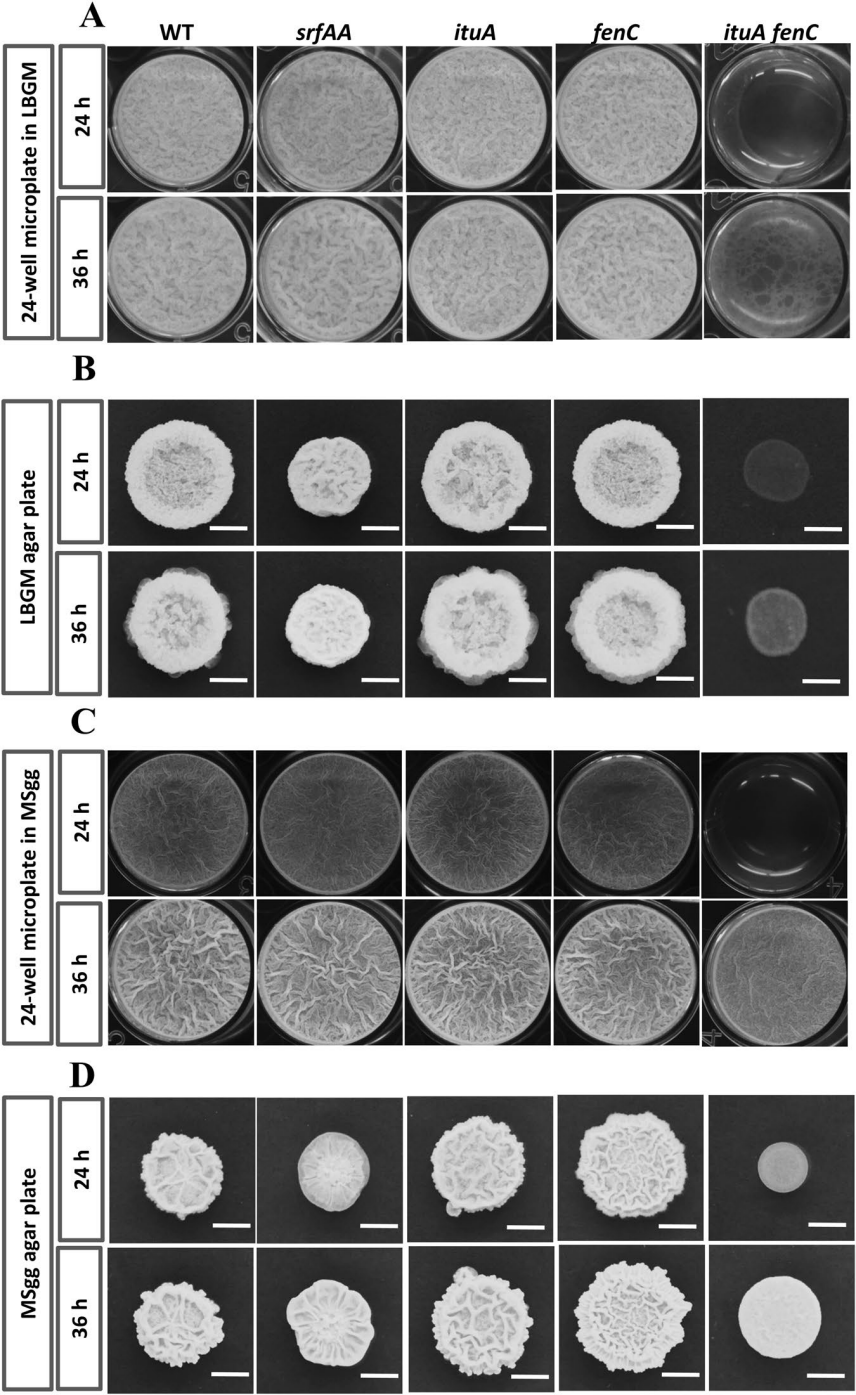
Either iturin A or fengycin is sufficient for biofilm formation. Pellicle formation on LBGM (**A**) and MSgg (**C**) liquid media and colony morphology on LBGM (**B**) and MSgg (**D**) plates was monitored for WT (Y6) and its derived mutants including *srfAA* (deficient in surfactin synthesis), *ituA* (deficient in iturin synthesis), *fenC* (deficient in fengycin synthesis) and *ituA fenC* (deficient in both iturin and fengycin synthesis). Representative photographs were taken after 24 or 36 h of incubation at 30 °C. The scale bar is 5 mm.

Bacteria often exist in the environment as cell communities called biofilms, which are required to effectively colonize plant roots and protect cells against unfavorable conditions^37,38^. Thus, biofilm formation is a prerequisite for effective *Bacillus* PGPR activity^22,39^. Surfactin is well known for its role in biofilm formation in the phylloplane and rhizosphere^22,40,41^. However, the contribution of the other LPs to biofilm formation is unclear. Therefore, we assessed the contribution of all three LPs produced by Y6 to this multicellular process by monitoring pellicle formation and colony morphology in biofilm stimulating media (LBGM and MSgg), where no evident growth defects were observed for all the strains tested (Fig. S7). As expected, WT (Y6) forms robust, wrinkled pellicles on LBGM (Fig. 6A) and MSgg (Fig. 6C) liquid medium. WT also forms complex colony patterns on both LBGM (Fig. 6B) and MSgg plates (Fig. 6D). The *srfAA* single mutant, which is defective in surfactin production, showed apparent defects in colony morphology on both LBGM and MSgg plates, but no noticeable defects in pellicle formation (Fig. 6). Mutation of *ituA* or *fenC* alone has no evident effects in biofilm formation. By contrast, the *ituA fenC* double mutant displays a flat and non-wrinkled biofilm architecture and an unstructured pellicle in comparison to the wild-type strain (Fig. 6), indicating iturin and fengycin, like surfactin, play an important but redundant role in facilitating biofilm formation.

### Either iturin or fengycin is sufficient for cell motility

**Figure 7:**
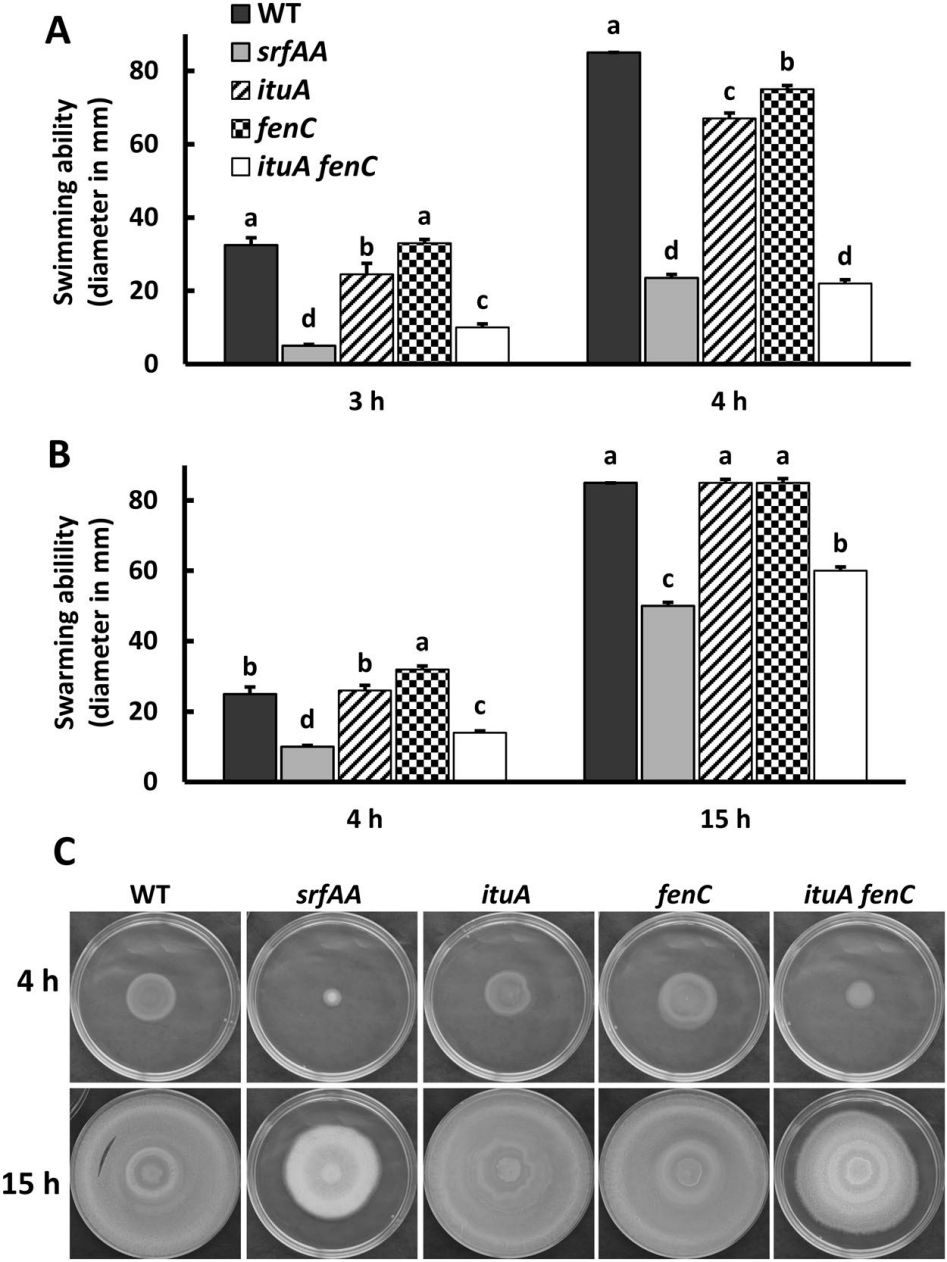
Either iturin or fengycin is sufficient for cell motility. (**A**) Swimming ability of Y6 (WT) and its derived mutants including *srfAA* (deficient in surfactin synthesis), *ituA* (deficient in iturin synthesis), *fenC* (deficient in fengycin synthesis) and *ituA fenC* (deficient in both iturin and fengycin synthesis) was tested. Five µl of LB preculture (OD_600_~0.4) was spotted onto LBGM plates (0.3% agar) and plates were incubated at 37 °C for 3 h or 4 h as indicated. (**B**) Swarming ability of the same strains as in panel (**A**) was evaluated. 5 µl of LB culture (OD_600_~0.4) were spotted on LBGM plates containing 0.7% agar and plates were incubated at 37 °C for 4 or 15 h. For both (**A**,**B**), data are expressed as the mean ± SD (n = 3). Significant differences among different strains at each timepoint tested are indicated as different letters on top of the data bars. The statistical analysis was determined by a Tukey’s Studentized Range (HSD) test: α = 0.05, n = 3. (**C**) Representative photographs to show the differences in colony swarming morphology among Y6 (WT) and its derived mutants at different time points (4 and 15 h).

Many bacteria move on surfaces towards nutrient-rich niches. In the rhizosphere, the ability of *Bacillus* to sense and move towards secreted plant compounds is critical for root colonization^42^. LPs and biosurfactants such as surfactin play important roles in the motility of some *Pseudomonas* and *Bacillus* isolates^43^. To understand the contribution of LPs to cell motility, we tested the surface motility of WT (Y6) and its derived mutants on LBGM medium plates prepared with varying levels of agar (Fig. S7). On “swim plates” (LBGM plates containing 0.3% agar)^44^, the wild-type (Y6) strain moved rapidly and completely spread over the whole plate within 4 h with a diameter of 85 mm. The *ituA* or *fenC* single mutants showed moderate defects in swimming with a diameter of 73 or 80 mm, respectively (Fig. 7A). In contrast, the *ituA fenC* double mutant almost completely lost its ability to swim with a diameter of 22 mm by 4 h, very similar to that of a surfactin-deficient strain (*srfAA*) (Fig. 7A). On plates to assess swarming motility (LBGM plates containing 0.7% agar)^44^, WT or either single mutant (*ituA* or *fenC*) was able to completely colonize the surface with a diameter of 85 mm by 15 h, whereas the *ituA fenC* double mutant showed a significant defect in swarming with a diameter of 60 mm by 15 h (Fig. 7B,C), close to that of *srfAA* mutant (50 mm in diameter by 15 h). These results suggest that in addition to surfactin, iturin and fengycin play important roles in surface movement, and either one is sufficient to facilitate cell motility.

## Discussion

Tomato bacterial wilt and banana *Fusarium* wilt are two destructive diseases to crops. Most chemical controls are ineffective against these diseases. Some lipopeptide (LP)-antibiotic producing *Bacillus* species offer a promising strategy in plant disease management. Here, we isolated two rhizosphere-associated *B. velezensis* strains (Y6 and F7) that exhibit potent antagonistic activity against bacterial and fungal pathogens in both laboratory and greenhouse settings. We demonstrated that the two LPs (iturin and fengycin) secreted by these two isolates are, chiefly if not solely, responsible for their antimicrobial activity. We then observed that expression of LP biosynthesis genes is upregulated and production of LPs is greatly stimulated in the presence of *R. solanacearum*. Modulation of LP production by pathogenic organisms may be a common natural phenomenon^32,45,46^. Indeed, the expression of LP genes including *ituC*, *fenD*, and *srfAA* in *B. amyloliquefaciens* D29 is significantly upregulated during interaction with *R. solanacearum*^45^ and in *B. amyloliquefaciens* Bk7 in response to *Pseudomonas fuscovaginae*^46^. Production of iturin and fengycin is strongly enhanced in *B. subtilis* 98S encountering fungal phytopathogens^32^. Interestingly, only *fenC* but no other LP genes is upregulated by the fungal pathogen *Rhizomucor variabilis*^47^. Thus, modulation of LP production might be a species-specific response between antagonist and pathogen. Although the triggering mechanism is largely unknown, it may involve the DegS/U two component sensing system, which is involved in the switch from a motile to sessile lifestyle and is thought to regulate lipopeptide production in *B. subtilis* NCD-2 and *B. velezensis* FZB42^48,49^.

The relative contribution of different LPs to antimicrobial activity may be dependent on the species of plant pathogen encountered. Our results showed that iturin and fengycin are functionally redundant in antagonism against *R. solanacearum*. By contrast, we observed that iturin, not fengycin, produced by Y6 plays the primary role, both *in vitro* and *in vivo*, in defense against the fungal pathogen Foc. Similar observations have been reported previously in other *Bacillus* isolates. In *B. velezensis* FZB42 and SQR9, bacillomycin D (a LP antibiotic related to iturin) is major contributor to antifungal activity against Foc while fengycin plays a minor role^9,12^. Fengycin has been observed to play an important role in antagonism against various fungal phytopathogens in *B. subtilis* UMAF6639 and EA-CB0015^50,51^. Additionally, in *B. subtilis* UMAF6639, iturin but not fengycin inhibits the pathogens *Pectobacterium carotovorum* and *Xanthomonas campestris*^31^.

Surfactin has been shown to have antagonistic activity *in vitro* and play protective roles in the rhizosphere^22,39,40^. We investigated the contribution of surfactin to the protective effects of the isolate Y6. Our results show that surfactin plays a minor role in antagonism against *R. solanacearum* (Fig. 4). We observed that fengycin and iturin production by Y6 is dramatically induced during interaction with *R. solanacearum* while surfactin levels remain low (Figs 2 and 3). Furthermore, surfactin is the least active antibacterial compound among all three LPs tested (Fig. S2).

The contribution of LPs to biocontrol outside of their direct antimicrobial activity is not well understood. We constructed LP deficient mutants and dissected the contribution of each individual LP to different biological processes. Surfactin may contribute to traits required for effective rhizosphere colonization such as biofilm formation and cell motility as reported previously^39,40,52,53^. Indeed, surfactin is important for biofilm formation, particularly on a solid surface (Fig. 6B,D), and is critical for cell motility (Fig. 7). Our results also show that iturin and fengycin contribute to biofilm formation and motility, as observed in *B. subtilis* 916^48^, *B. velezensis* LL3 and SQR9^12,54^. Thus, we propose that the biocontrol activity of Y6 is the result of coordinated actions of all three LPs.

Collectively, this study demonstrates that two LP-producing *B. velezensis* isolates (Y6 and F7) have potent antagonistic activity against *R. solanacearum* and Foc under both laboratory and greenhouse conditions. LP production in these two isolates is strongly stimulated in the presence of the pathogenic strain *R. solanacearum* and is critical for antagonism and colonization in controlled-environmental conditions. Most importantly, these two isolates, as potential biocontrol agents, may provide an effective strategy to combat plant pathogens in the target environment.

## Materials and Methods

### Isolation of antagonistic *Bacillus* species

Samples were collected from the rhizosphere soil of tomato plants in Yuejin Farm, Guangzhou, China (23° 08′N 113° 16′E). *Bacillus* spp. were isolated as reported previously^55^. Briefly, a soil sample (10 g) was shaken in 90 ml of sterilized water for 30 min, heated for 30 min at 80 °C, serially diluted and then spread over lysogeny broth (LB)^56^ plates. Single bacterial colonies were streaked onto fresh LB plates after 48 h of incubation at 30 °C. Frozen stocks of the purified colonies were prepared using 15% glycerol and kept at −80 °C for further study.

### Antibacterial activity test

The antibacterial activity of these isolates and their derived mutants against *R. solanacearum* GMI1000^57,58^ was evaluated using an optimized spot-on-lawn assay. 200 μl of *R. solanacearum* cell culture (OD_600_ ~0.4) grown in Casamino acid-Peptone-Glucose (CPG, 0.1% peptone, 0.01% casamino acids, 0.05% glucose) was mixed with 4 ml of 0.7% CPG soft agar and directly poured onto a CPG plate (1.5% agar). Plates were dried for 50 min and a well (5 mm in diameter) was made in the center of each plate, and 50 μl of each *Bacillus* strain (OD_600_~0.4) grown in LB medium was added into each well. Antagonistic activity was evaluated based on the inhibition zone after 24 h incubation at 30 °C. Antibacterial activity of the methanol extracts of Y6 and F7 was tested using an agar diffusion assay. Briefly, 200 μl of *R. solanacearum* CPG culture (OD_600_ ~0.4) was mixed with 4 ml of 0.7% CPG soft agar and directly poured onto a CPG plate (1.5% agar), and 5 µl of methanol extract was added into each well (5 mm in diameter). The methanol extracts (5 µl) of *B. megaterium* X3 (Genbank accession number KJ526881) and methanol alone were used as controls. The inhibition zone was measured after 24 h of incubation at 30 °C. The experiments were performed at least three times.

### Antifungal activity test

We performed two assays, a plate confrontation assay and a spot-on-lawn assay, to test activity of the isolates (Y6 and F7) and derived mutants against common fungal pathogens including *Fusarium oxysporum* f.sp*. cubense* 4 strain XJZ2 (Foc4, GenBank accession number JX090598)^59^, *F. oxysporum* f.sp. *cucumerinum* (isolated from *cucumerium* rhizosphere, no accession number available yet), and *Colletotrichum gloeosporioides* (Genbank accession number MWUF00000000)^60^. In the plate confrontation assay, the fungi were cultivated on potato-dextrose-agar plates (PDA, 20% potato infusion, 2% dextrose, and 1.5% agar) at 30 °C for 5 days. A 5-mm-diameter block of mycelium agar was cut and transferred into the center of a fresh PDA plate. After 1 day of incubation, 3 µl of *Bacillus* spp. cells (F7, Y6, or mutants derived from Y6) (OD_600_ ~0.4) grown in LB medium was spotted on the PDA plate 2.5 cm away from the center, where the mycelium agar block was placed. The antifungal activity was evaluated by measuring the diameter of the inhibition zone (the distance between the Foc mycelium and the bacterial colony) after 7 days of incubation at 30 °C.

We also developed a spot-on-lawn assay, which is more sensitive compared to the plate confrontation assay and only requires one-day incubation instead of seven days. Briefly, the fungal hyphae Foc4 were streaked and inoculated with 5 ml of PDA broth. After 2-day incubation at 30 °C with 180 rpm shaking, 50 μl of fungal culture was re-inoculated into 5 ml of fresh PDA broth, incubated for additional 12 h, filtered using a cheesecloth to remove hyphae, and then the concentration of spores was determined using a hemocytometer under a light microscope. Fifty µl of a spore suspension (~10^6^ spores ml^−1^) was mixed with 4 ml of 0.7% soft PDA agar and directly poured onto a PDA plate (1.5% agar). Plates were dried for 50 min and a well (5 mm in diameter) was made in the center of each plate, and 50 µl of *Bacillus* spp. cells (OD_600_ ~0.4; F7, Y6, or mutants derived from Y6) grown in LB medium was added into each well. The antifungal activity was evaluated by measuring the diameter of the inhibition zone (mm) after 24 h of incubation at 30 °C. The experiments were performed at least three times.

### Lipopeptide (LP) extraction from the isolated strains

LPs were extracted according to previous protocol^61^. Briefly, 150 μl of Y6 or F7 cell culture (OD_600_ ~0.4) was added into 15 ml of fresh LB medium. After 2 days of incubation at 30 °C with 180 rpm shaking, 10 ml of cell culture was spun down by centrifugation for 5 min at 14000 × g, and the cell-free supernatant was collected and subjected to filtration using 0.22-μm filters, and then loaded into a C_18_ syringe cartridge column (Bond Elut C_18,_ Agilent, USA). The column was washed with 10 ml of ddH_2_O and the LP compounds were eluted with 2 ml of methanol.

To understand the influence of pathogen on LP production, we extract LPs from the inhibition zone (Fig. 1A)^32^. 200 μl of *R. solanacearum* cell culture (OD_600_ ~0.4) grown in CPG medium was mixed with 4 ml of 0.7% CPG soft agar and directly poured onto a CPG plate (1.5% agar). Plates were dried for 50 min and a well (5 mm in diameter) was made in the center of each plate, and 50 μl of *Bacillus* isolates (Y6 or F7) (OD_600_ ~0.4) grown in LB medium was added into the well. After the inhibition zone became evident with 24 h incubation at 30 °C, a 300 mg of agar sample was harvested from the inhibition zone, mixed with 1 ml of acetonitrile/water (1:1 v/v), and sonicated for 30 s followed by centrifugation and filtration. The supernatant was collected from acetonitrile/water extract^32^. As a control, LPs were isolated in a similar way but without *R. solanacearum* in the top soft agar layer. Agar sample (300 mg) was collected around the well (2–4 mm).

### Identification and quantification of LPs by UPLC–MS

Both methanol and acetonitrile/water extracts were analyzed by reverse phase Ultra-Performance Liquid Chromatography coupled with a triple quadrupole MS (UPLC-MS) (Waters, Acquity, XEVO-TQD). The lipopeptide compounds were identified based on the mass-to-charge ratio (m/z) and quantitated based on the standard curves generated using commercial LP standards (Sigma-Aldrich, USA). The column temperature was maintained at 40 °C and a gradient elution with (A) acetonitrile (containing 0.1% formic acid) and (B) water (containing 0.1% formic acid) was used. The gradient program was used as follows: 0–0.5 min, 40% A; 0.5–3.5 min, 40–80% A; 3.5–4.0 min, 80% A; 4.0–6.0 min, 80–95% A; 6.0–7.0 min, 95–98% A. The flow rate was set at 0.4 ml min^−1^. The Electrospray Ionization (ESI) source was set in positive ionization mode with 3.26 kV of capillary voltage, and the source temperature was maintained at 150 °C. Nitrogen flow was 600 L h^−1^. Argon flow was 50 L h^−1^.

### Construction of mutants deficient in LP synthesis

In initial studies, we determined that Y6 is sensitive to spectinomycin (*spc*; 100 μg ml^−1^), kanamycin (*kan*; 15 μg ml^−1^), chloramphenicol (*cat*; 10 μg ml^−1^), tetracycline (*tet*; 5 μg ml^−1^), and macrolide lincosoamide-streptogramin B (*mls*; contains 1 μg ml^−1^ erythromycin and 25 μg ml^−1^ lincomycin) antibiotics, whereas F7 was sensitive to *kan, cat* and *mls*, but resistant to *spc*. Therefore, strain Y6 was selected for mutant construction. The *srfAA:mls*, *ituA:mls*, and *fenC:spc* single mutants and the double mutants *ituA:mls fenC:spc* and *ituA:mls fenB:spc* were generated by replacing the coding region with a resistance cassette using long flanking homology PCR (LFH-PCR) followed by DNA transformation as previously described^62^ (Table S1). Specific primers used for PCR amplification were designed based on the gene sequences of *B. velezensis* FZB42 (*srfAA* and *ituA*) and DSM7 (*fenC*) (Table S2).

### Swarming and swimming motility assays

Swimming and swarming motility of Y6 and its derived mutants were tested using standard protocols with minor modification^63,64^. LBGM plates containing 0.7% (swarming) or 0.3% agar (swimming) were dried in a laminar flow hood for 30 min and then 5 μl of LB precultures (OD_600_ ~0.4) was spotted on the center of each plate. The plates were then dried for another 15 min and incubated overnight at 37 °C.

### Biofilm formation assay

To evaluate the contribution of the LP compounds to biofilm formation, we monitored colony morphology on LBGM and MSgg plates and pellicle formation on LBGM and MSgg liquid media. For colony morphology analysis, 3 μl of LB preculture (OD_600_ ~0.4) was spotted onto LBGM or MSgg agar plates, which were dried for 30 min in a laminar airflow prior to spotting, and incubated at 30 °C for up to 36 h. To monitor pellicle formation^65^, 10 μl of LB precultures (OD_600_ ~0.4) was inoculated into 2.5 ml of LBGM or MSgg medium in a 24 well plate and incubated at 30 °C for up to 36 h^65,66^.

### Additional methods

Additional methods are described in the Supplementary Information, including plant pot experiments, identification of the isolates Y6 and F7 by phylogenetic analysis, minimal inhibition concentrations (MIC) determination of LPs and the extracts from the isolates, RNA extraction and quantitative PCR (qPCR), growth curves, and spore germination assay.

### Data availability

All data generated during this study are included in this article (and its Supplementary Information file).

## Electronic supplementary material

Supplemental information
