# Supplementary material for: Antagonism of Two Plant-Growth Promoting Bacillus velezensis Isolates Against Ralstonia solanacearum and Fusarium oxysporum
Source: Sci Rep. 2018 Mar 12;8:4360. doi: 10.1038/s41598-018-22782-z (PMC5847583; doi:10.1038/s41598-018-22782-z)
Supplement: Supplementary file 1 — Supplemental information [file 41598_2018_22782_MOESM1_ESM.pdf]

Antagonism of Two Plant-Growth Promoting *Bacillus velezensis* Isolates Against *Ralstonia solanacearum* and *Fusarium oxysporum*

Yu Cao[1†], Hualiang Pi[2†], Pete Chandrangsu[2], Yongtao Li[1], Yuqi Wang[1,3], Han Zhou[1], Hanqin Xiong[1,4], John D. Helmann[2*] and Yanfei Cai[1, 2*#]

College of Natural Resources and Environment, South China Agricultural University, Guangzhou, 510642, PR China[1]; Department of Microbiology, Cornell University, Ithaca, NY 14853-8101, USA[2]; College of Environmental Science and Engineering, Guangzhou University, Guangzhou, 510006, PR China[3]; Guangzhou Daodong New Energy Co. Ltd, 510670, PR China[4].

†These two authors contributed equally to this work.

Methods
References
Table S1 Strains used in this study
Table S2 Oligonucleotides used in this study
Table S3 Lipopeptide compounds (LPs) produced by the two *B. velezensis* isolates.
Fig. S1. Identification of Y6 and F7 by phylogenetic analysis.
Fig. S2. Inhibitory effects of standard LPs and LP extracts from the isolates against Rs.
Fig. S3. Crosstalk between iturin A and fengycin production.
Fig. S4. Antifungal activity of *B. velezensis* Y6 and F7 against different fungal pathogens.
Fig. S5. Antifungal activity of Y6 and its derived mutants against Foc.
Fig. S6. Inhibitory activity of LP standards and Y6 extract against Foc spores.
Fig. S7. Cell growth of Y6 and its derived mutants in various media.

Methods

Plant pot experiments

The biocontrol efficacy of the two isolates Y6 and F7 against tomato bacterial wilt was determined under greenhouse conditions. Three treatments were included as follows: control (CK, *R. solanacearum* only), Y6 (*R. solanacearum* + Y6), F7 (*R. solanacearum* + F7). Tomato seeds (*Lycopersicon esculentum* Miller) were surface-sterilized with 70% ethanol for 30 s and 5% sodium hypochloride for 15 min followed by three washes with sterile water for 15 min each time. The surface-sterilized seeds were incubated in pots with non-sterile local soil (pH 5.4, soil organic matter 23.1 g kg^{-1}, soil total nitrogen 1.0 g kg^{-1}, soil total phosphorus 5.8 g kg^{-1}), and the pots were then placed in an artificial climate chamber (PQX-450R-22HM), which is maintained at 25-32˚C with relative humidity from 65% to 80%. After one month, the tomato seedlings were transplanted into pots containing 1.5 kg of the non-sterile local soil. Seven days after transplanting, the soil used for Y6 or F7 treatment was drenched with 50 ml of bacterial suspension of Y6 or F7 in sterile water (～10^6 cells per g of soil). The bacterial suspension was prepared using cell pellets harvested from 50 ml of LB cell culture (OD_{600} ~1.0) by centrifugation. Two days later, 30 ml of *R. solanacearum* cell suspension, which was obtained from 30 ml of CPG cell culture (OD_{600} ~1.0) by centrifugation and resuspension in sterile water, was drenched into each pot to infect the plants (～10^7 cells per g of soil). The pots were then placed back into the artificial climate chamber with day/night cylce (16/8 h), at day 30˚C and night 28˚C with relative humidity 100%. The whole set of experiment was performed twice in both spring (Jan.-Feb.) and autumn (Sep. to Oct.) of 2015, with 24 tomato plants used for each group. The wilt incidence (WI) was calculated on the 30th day after transplanting. The disease severity index (DSI) was recorded as follows[1]: 0 = no wilt symptoms, 1 = wilt symptoms on 1-25% of the leaves, 2 = wilt symptoms on 26-

50% of the leaves, 3 = wilt symptoms on 51-75% of the leaves, and 4 = wilt symptoms on more than 76% of the leaves.

To understand the contribution of iturin and fengycin in the biocontrol efficacy against banana *Fusarium* wilt under controlled-environmental conditions, banana pot experiments were performed under greenhouse conditions using Micropropagated Cavendish banana seedling '*Brazilian*', the *F. oxysporum (*Foc) susceptible variety. Two control groups were included as follows: CK1 (no Foc): banana seedlings with four or five leaves and approximately 20 cm in height were directly planted into pots. CK2 (Foc, GenBank accession number JX090598): prior to planting into pots, the roots of the banana seedlings were immersed in Foc (～10^6 spores ml^{-1}) for 30 min. Four treated groups were included: Foc + WT (Y6), Foc + *ituA* single mutant, Foc + *fenC* single mutant and Foc + *ituA fenC* double mutant. The roots of the banana seedlings were immersed firstly in Foc for 30 min, then planted into pots. After two days, the plants were watered with 50 ml of cell suspension (OD_{600} ~1.0) of Y6 and its derived mutants around the roots to reach a final concentration of ～10^6 cells per gram of soil. The disease severity index (DSI) was recorded as follows [2], 1 = healthy, 2 = slight chlorosis and wilting with no petiole buckling, 3 = moderate chlorosis and wilting with some petiole buckling and/or splitting of leaf bases, 4 = severe chlorosis, wilting, petiole buckling and dwarfing of the newly emerged leaf, and 5 = dead. Wilt incidence (WI) of the banana plants was monitored every 3 days after transplantation. Wilt incidence and biocontrol efficacy of both tomato and banana pot experiments were calculated according to the following formula [1,2]:

$$\text{Wilt incidence (WI)} = \frac{\sum \text{(number of infected plants} \times \text{DSI)}}{\text{Total number of plants investigated} \times \text{highest DSI}} \times 100\%$$

$$\text{Biocontrol efficacy} = \frac{\text{WI (pathogen only control)} - \text{WI (treated group)}}{\text{WI (pathogen only control)}} \times 100\%$$

Identification of the isolates Y6 and F7 by phylogenetic analysis

Genomic DNA was extracted from the two isolates using a DNeasy® blood & tissue kit from Qiagen (Hilden, Germany; Cat # 69506). The 16S *rRNA* gene was amplified by PCR using the extracted genomic DNA as a template and a specific primer set (27F and 1492R) [3]. The gene sequence was then determined by DNA sequencing. The DNA sequence of *gyrA* was obtained from Illumina DNA sequencing of these two isolates (unpublished data). To identify these two isolates, phylogenetic analysis was conducted using a wide range of *Bacillus* species based on 16S *rRNA* and *gyrA* gene sequences. The phylogenetic trees were constructed using a neighbor-joining method.

Minimal inhibition concentrations (MIC) determination of LPs and the extracts from the isolates

The MICs of the LP standards including surfactin, iturin and fengycin (Sigma-Aldrich, USA) and the acetonitrile/water extracts from the isolates were determined by a microtiter broth dilution assay. Briefly, *R. solanacearum* preculture (OD_{600} ~0.4) was inoculated with a 1% ratio into fresh CPG broth amended with various concentrations of the standards or LP extracts. Cell growth (OD_{600}) was monitored every 15 min for 18 h using a Bioscreen growth analyser (Growth Curves USA, Piscataway, NJ) at 30°C with continuous shaking. The MIC was defined as the minimum antibiotic concentration that prevents bacterial growth (final $OD_{600} < 0.01$). The experiments were performed with three biological replicates.

RNA extraction and quantitative PCR (qPCR)

To evaluate the influence of the pathogen *R. solanacearum* on expression of LP biosynthesis genes, *Bacillus* cells (Y6 or F7) were harvested from CPG plates co-cultivated with *R. solanacearum* as described below. Two hundred µl of *R. solanacearum* preculture (OD_{600} ~0.4) was mixed with 4 ml

of 0.7% CPG soft agar and directly poured onto a CPG plate (1.5% agar). Plates were dried for 50 min and a well (6.5 mm) was made on the center of each plate. Fifty µl of LB preculture (OD_{600} ~0.4) of Y6 or F7 was added into each well. After 30 h of incubation at 30°C, *Bacillus* cells (Y6 and F7) were collected, washed with PBS buffer to remove any remaining agar and the cell pellets were harvested by centrifugation. For the control, *Bacillus* cells were harvested in a similar manner but without *R. solanacearum* present in the top soft agar layer. To monitor the transcription changes of LP genes in Y6 and its derived mutant strains during interaction with *R. solanacearum,* cells were collected in a same way as described above. All samples were then subjected to total RNA extraction using RNeasy Mini Kit following the manufacturer's instructions (Qiagen Sciences, Germantown, MD). All RNA samples were treated with Turbo-DNA free™ DNase (Ambion™) and 200 ng of total RNA from each sample was subjected to cDNA synthesis followed by qPCR using iQ SYBR green supermix in an Applied Biosystems 7300 Real Time PCR System. Primers used in this study are listed in Table S2. The 23S *rRNA* gene was served as an internal control. The relative gene expression changes were calculated using the $2^{-\Delta\Delta C_T}$ method [4].

Growth curves

To monitor growth, cells were grown to an OD_{600} of 0.4 in LB medium. Aliquots (2 µl) were inoculated to 200 µl of different media as indicated in a Bioscreen 100-well microtiter plate. The media used include LB, CPG, LBGM (LB with 1% glycerol (v/v) and 0.1 mM manganese) [5] and MSgg (5 mM potassium phosphate, pH 7; 100 mM morpholinepropanesulfonic acid, pH 7; 2 mM $MgCl_2$, 700 µM $CaCl_2$, 50 µM $MnCl_2$, 50 µM $FeCl_3$, 1 µM $ZnCl_2$, 2 µM thiamine, 0.5% glycerol, 0.5% glutamate, 50 µg ml^{-1} tryptophan, 50 µg ml^{-1} phenylalanine, and 50 µg ml^{-1} threonine) [6]. Growth was monitored spectrophotometrically (OD_{600}) every 15 min for 24 h using a Bioscreen growth analyzer (Growth

Curves USA, Piscataway, NJ) at 30°C with continuous shaking. Data shown were representative growth curves and experiments were performed three times with three biological replicates each time.

Spore germination assay

Antifungal activity of LP standards and Y6 extract against Foc was conducted using a spore germination assay according to the standard protocol [7]. Fungal cells were inoculated in Czapek–Dox Medium (sucrose 30 g L^{-1}, sodium nitrate 3 g L^{-1}, dipotassium phosphate 1 g L^{-1}, magnesium sulphate 0.5 g L^{-1}, potassium chloride 0.5 g L^{-1}, ferrous sulphate 0.01 g L^{-1} (pH 7.3)) for two days at 30°C with continuous shaking. The culture was filtered using a cheesecloth and the spores were harvested by centrifugation and resuspended in fresh PDB medium with a final concentration 1×10^6 spores ml^{-1}. Fifty µl of the suspension was mixed with various concentrations of the LP standards or Y6 extract and incubated for 12 h at 30°C. The extent of spore germination was evaluated based on five randomly selected views under a light microscope. Experiment was performed in triplicate. Data are expressed as the average percentage of spores germinated in the treated group compared to the untreated control.

References

1 Chen, Y. *et al.* Biocontrol of tomato wilt disease by Bacillus subtilis isolates from natural environments depends on conserved genes mediating biofilm formation. *Environmental microbiology* **15**, 848-864, doi:10.1111/j.1462-2920.2012.02860.x (2013).

2 Ploetz, R. C., Haynes, J. L. & Vazquez, A. Responses of new banana accessions in South Florida to Panama disease. *Crop Protection* **18**, 445-449, doi:Doi 10.1016/S0261-2194(99)00043-5 (1999).

3 Turner, S., Pryer, K. M., Miao, V. P. & Palmer, J. D. Investigating deep phylogenetic relationships among cyanobacteria and plastids by small subunit rRNA sequence analysis. *J Eukaryot Microbiol* **46**, 327-338 (1999).

4 Livak, K. J. & Schmittgen, T. D. Analysis of relative gene expression data using real-time quantitative PCR and the 2(-Delta Delta C(T)) Method. *Methods* **25**, 402-408, doi:10.1006/meth.2001.1262 (2001).

5 Shemesh, M. & Chai, Y. A combination of glycerol and manganese promotes biofilm formation in Bacillus subtilis via histidine kinase KinD signaling. *Journal of bacteriology* **195**, 2747-2754, doi:10.1128/JB.00028-13 (2013).

6 Branda, S. S., Gonzalez-Pastor, J. E., Ben-Yehuda, S., Losick, R. & Kolter, R. Fruiting body formation by Bacillus subtilis. *Proc Natl Acad Sci U S A* **98**, 11621-11626, doi:10.1073/pnas.191384198 (2001).

7 Das, K., Tiwari, R. K. S. & Shrivastava, D. K. Techniques for evaluation of medicinal plant products as antimicrobial agent: Current methods and future trends. *Journal of Medicinal Plants Research* **4**, 104-111 (2010).

Table S1 Strains used in this study

Strain	Genotype	Reference
168	*B. subtilis*	Lab stock
X3	*B. megaterium*	Lab stock
HB19424	*B. velezensis F7*	This study
HB19425	*B. velezensis Y6*	This study
HB19453	*srfAA:mls*	This study
HB19454	*ituA:mls*	This study
HB19458	*fenB:spc*	This study
HB19459	*fenC:spc*	This study
HB19460	*ituA:mls fenB:spc*	This study
HB19461	*ituA:mls fenC:spc*	This study

Note: all the mutants were constructed in *B. velezensis* Y6 background unless specified otherwise.

Table S2 Oligonucleotides used in this study

No	Name	Sequence
6715	*ituA*-up-fwd	CAAGCTGTGGAACGTGTCAT
6716	*ituA*-up-rev (mls)	GAGGGTTGCCAGAGTTAAAGGATCGAAGCTGTTTAGCCTCGCAA
6717	*ituA*-down-fwd (mls)	CGATTATGTCTTTTGCGCAGTCGGCAAACGAGGTGCTCCAATGTC
6718	*ituA*-down-rev	TGAAACAGCGTATCGGTTGC
6719	*srfAA*-up-fwd	GCAAGGAGAAAGCCTTATGC
6720	*srfAA*-up-rev (mls)	GAGGGTTGCCAGAGTTAAAGGATCCTCCCTCTATGTCCCTAACTC
6721	*srfAA*-down-fwd (mls)	CGATTATGTCTTTTGCGCAGTCGGCGTGTATGCACTCACACCGAT
6722	*srfAA*-down-rev	GGCTGCTCCAATATTGTTCC
6723	*fenB*-up-fwd	CATACGGAACGGATGTTCATG
6724	*fenB*-up-rev (spec)	CGTTACGTTATTAGCGAGCCAGTCATACTTGATAGTGCTTGAGCG
6725	*fenB*-down-fwd (spec)	CAATAAACCCTTGCCCTCGCTACGCAGCAACCGGAAATACAAGAT
6726	*fenB*-down-rev	CTCAAGTTCGCCATTTGGTT
6727	*fenC*-up-fwd	AGAGACGGGTGAATTATTGCT
6728	*fenC*-up-rev (spec)	CGTTACGTTATTAGCGAGCCAGTCATGTCACTCAGATCTTCCAGC
6729	*fenC*-down-fwd (spec)	CAATAAACCCTTGCCCTCGCTACGCGCTGTCTTATATGCAGGAAG
6730	*fenC*-down-rev	AACAGTCTGCTGTTCAAAGC
4368	23S-RT-fwd	AAAGGCACAAGGGAGCTTGACTGC
4369	23S-RT-rev	ATGAGCCGACATCGAGGTGCCAAA
6731	*srfAB-RT-fwd*	AAAGGTGTATGCACTCACACCG
6732	*srfAB-RT-rev*	ATGTCCGGATCAGCTCATTGAC
6739	*ituC-RT-fwd*	TCTTCGTTCAGACCAGCTCATG
6740	*ituC-RT-rev*	GCATTGTAGTTCAGCCTCAGCT
6741	*fenD-RT-fwd*	AAAGGAATCACAGGCTTACGCC
6742	*fenD-RT-rev*	CACTGACACGTTCTGGCTGAT
6743	*27F*	AGAGTTTGATCMTGGCTCAG
6744	*1492R*	GGTTACCTTGTTACGACTT

Table S3 Lipopeptide compounds (LPs) produced by the two *B. velezensis* isolates.

LPs	Isoforms	Molecular mass $[M+H]^+$	LPs produced by Y6 (μg ml^{-1})	LPs produced by F7 (μg ml^{-1})
iturin	C14	1043.7	13.3±1.0	10.6±2.6
	C15	1057.6	32.7±2.6	2.4±0.2
	C16	1071.7	8.8±1.1	7.0±0.6
Total iturin			54.9±4.8	20.0±3.5
fengycin	C12	1435.9	4.7±1.1	4.6±0.6
	C13	1449.9	14.2±3.0	10.3±3.7
	C14	1464.0	45.6±12.3	32.3±8.5
	C15	1478.0	69.8±18.3	36.2±12.6
	C16	1492.0	16.0±6.5	6.5±1.0
	C17	1506.0	25.1±6.6	8.8±3.1
Total fengycin			175.4±47.8	98.6±29.6
surfactin	C12	994.7	3.0±0.3	2.3±0.3
	C13	1008.7	9.8±1.1	9.9±1.8
	C14	1022.7	13.6±1.2	12.9±1.8
	C15	1036.8	10.5±0.4	11.5±1.9
	C16	1050.8	1.5±0.3	1.2±0.3
Total surfactin			38.5±3.4	37.8±3.4
Total LPs			258±56	156±39

Note: 10 ml of Y6 or F7 cell culture was grown at 30°C in LB medium for 48 h, harvested and subjected to UPLC-MS analysis. LP quantification was performed based on the standard curves of the commercial standards. Data are expressed as the mean ± SD (n = 3).

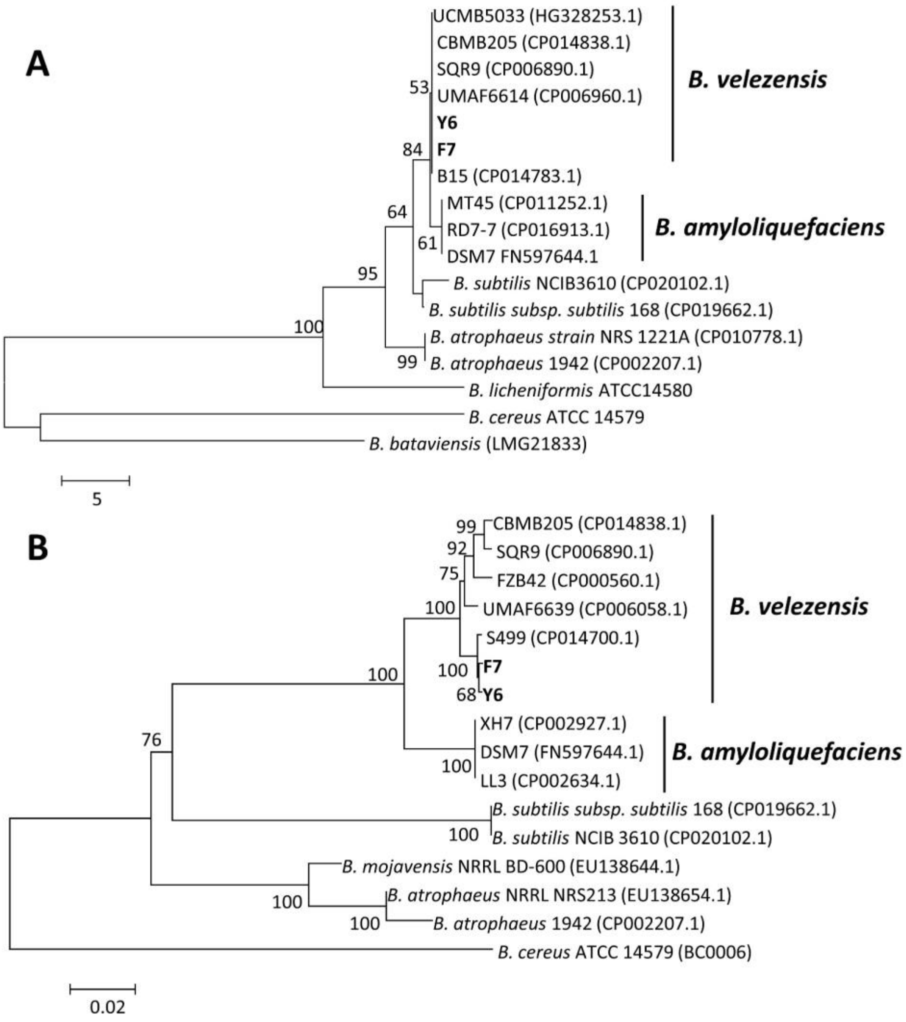

Fig. S1. Identification of Y6 and F7 by phylogenetic analysis.

The phylogenetic analysis was based on 16S *rRNA* (A) and *gyrA* (B) gene sequences. The phylogenetic trees were constructed using a neighbor-joining method. Only above 50% of bootstrap, based on 1000 replications, are indicated next to the branches. The scale bar, 5 (A) or 0.02 (B), indicates substitutions per nucleotide position. Both Y6 and F7 are clustered with some subspecies *plantarum* of *B. velezensis* such as S499 and UMAF6614, indicating that the two isolates are closely related to *B. velezensis subsp. plantarum*.

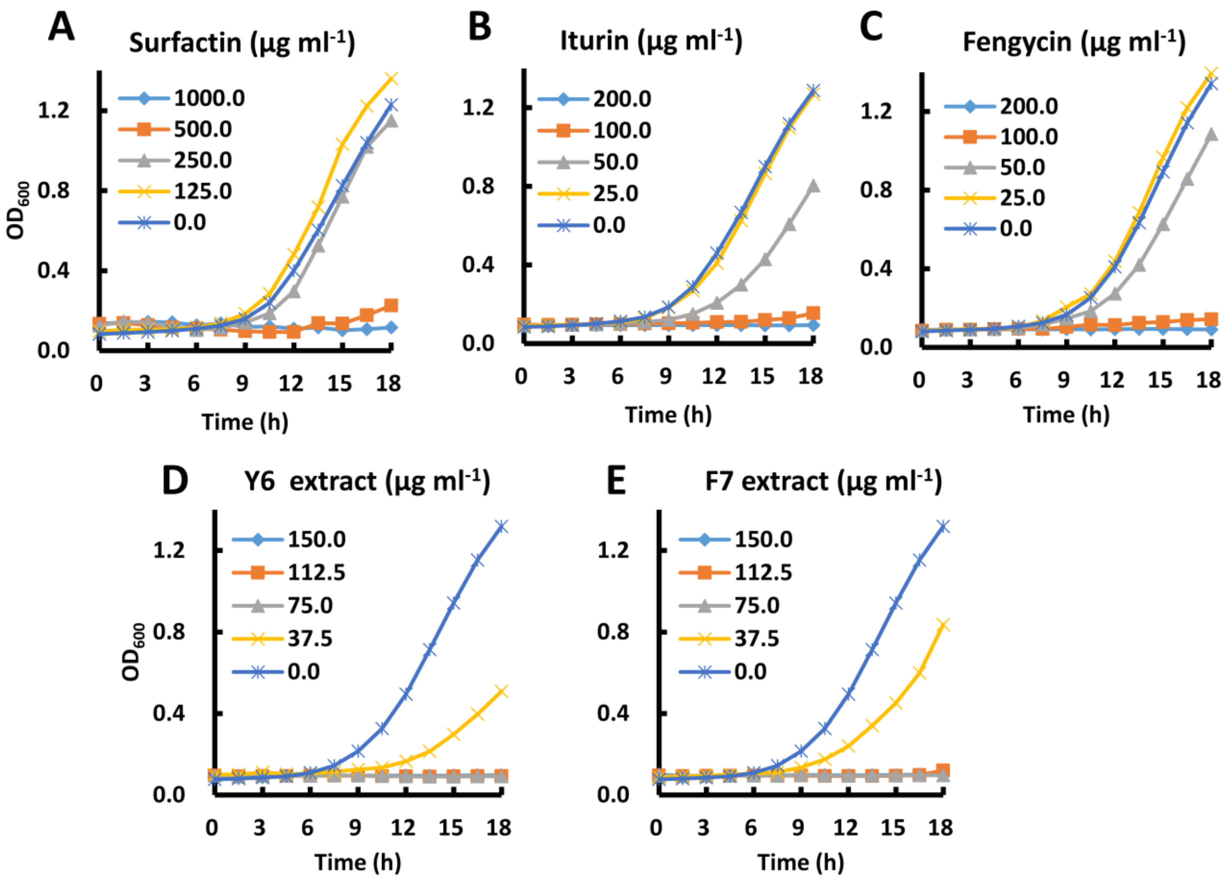

Fig. S2. Inhibitory effects of standard LPs and LP extracts from the isolates against Rs.

The MICs of the LP standards including surfactin, iturin and fengycin (Sigma-Aldrich, USA) and the acetonitrile/water extracts from the isolates were determined by a microtiter broth dilution assay. Briefly, *R. solanacearum* preculture (OD_{600} ~0.4) was inoculated with a 1% ratio into fresh CPG broth amended with various concentrations of the standards or acetonitrile/water extracts. Cell growth (OD_{600}) was monitored every 15 min for 18 h using a Bioscreen growth analyser (Growth Curves USA, Piscataway, NJ) at 30°C with continuous shaking. The MIC was defined as the minimum antibiotic concentration that prevents bacterial growth (final $OD_{600} < 0.01$). The experiments were performed with three biological replicates.

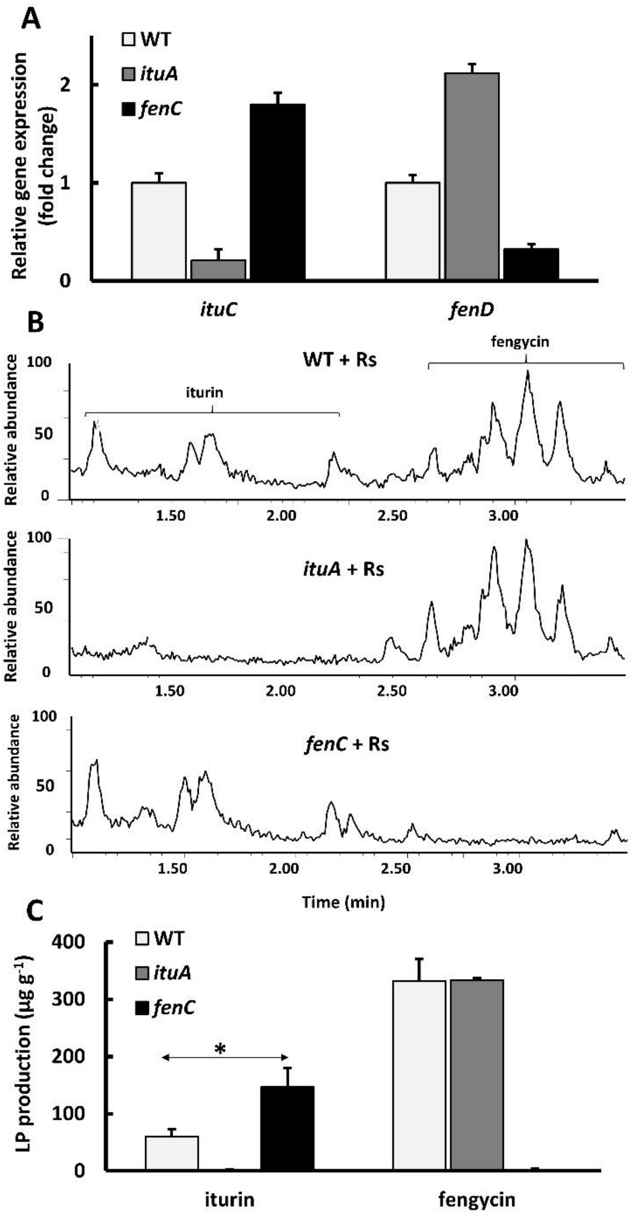

Fig. S3. Crosstalk between iturin A and fengycin production.

A. Expression of the LP biosynthesis genes (*ituC* and *fenD)* was monitored by qPCR in WT (Y6), *ituA*, and *fenC* single mutants in the presence of the pathogen *R. solanacearum* (*Rs*). RNA extraction and qPCR were performed as described in Materials and Methods. The relative gene expression (in fold change) was calculated using the $2^{-\Delta\Delta C_T}$ method.

B. Representative chromatograms illustrate the differences in LP production by strains Y6, *ituA*, and *fenC* single mutants in the presence of the pathogen *R. solanacearum*.

C. LP compounds were extracted from the inhibition zone (Fig. 4) and quantified by UPLC-MS analysis as described in Materials and Methods. Briefly, after the inhibition zone against *R. solanacearum* (Rs) became evident with 24 h incubation at 30°C, a 300 mg of agar sample was harvested from the inhibition zone, mixed with 1 ml of acetonitrile/water (1:1 v/v), and sonicated for 30 s followed by centrifugation and filtration. The supernatant was collected as the acetonitrile/water extract and subjected to UPLS-MS analysis. Significant difference between WT and *fenC* single mutant is determined by two-tailed t-test as indicated: *P < 0.05, n = 3.

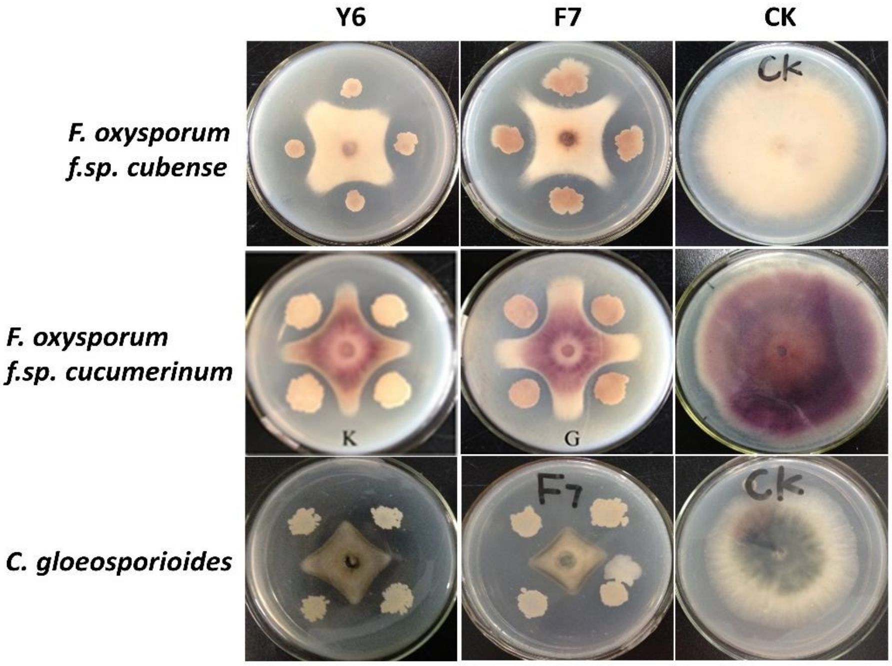

Fig. S4. Antifungal activity of *B. velezensis* Y6 and F7 against different fungal pathogens.

A plate confrontation assay was conducted to test antifungal activity of the isolates against common fungal pathogens (*Fusarium oxysporum* f.sp. *cubense*, *F. oxysporum* f.sp. *cucumerinum*, and *Colletotrichum gloeosporioides*). The pathogenic fungal cells were cultivated on potato-dextrose-agar plates (PDA, 20% potato infusion, 2% dextrose, and 1.5% agar) at 30°C for 5 days. A 5-mm-diameter block of mycelium was cut and transferred into the center of a fresh PDA plate. After 1 day of incubation, 3 µl of *Bacillus* cells (F7 or Y6) (OD_{600} ~0.4) grown in LB medium was spotted on the PDA plate 2.5 cm away from the center, where the mycelium block was placed. CK serves as a negative control without addition of any antagonistic agent. The antifungal activity was evaluated after 7 days of incubation at 30°C. The experiments were performed at least three times. Representative photographs are shown.

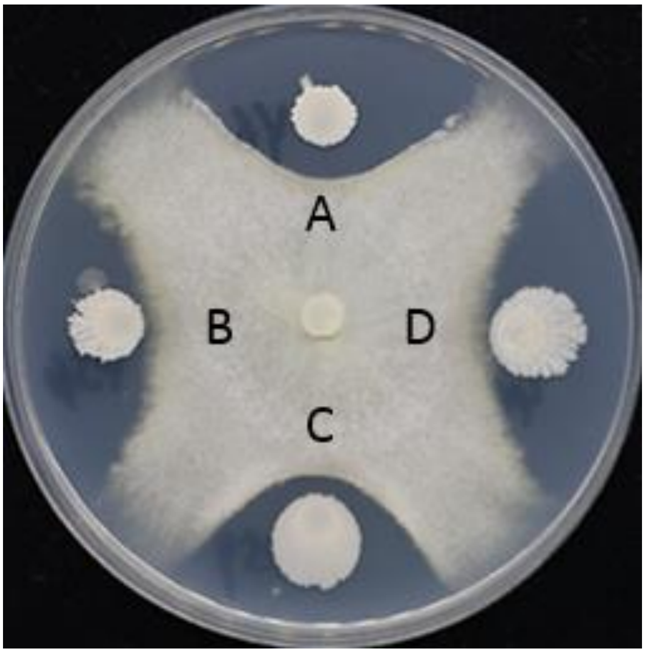

Fig. S5. Antifungal activity of Y6 and its derived mutants against Foc.

The antifungal activity of Y6 and its derived mutants against Foc was tested using a plate confrontation assay. Briefly, the pathogenic fungal cells were cultivated on potato-dextrose-agar plates (PDA, 20% potato infusion, 2% dextrose, and 1.5% agar) at 30ºC for 5 days. A 5-mm-diameter block of mycelium was cut and transferred into the center of a fresh PDA plate. After 1 day of incubation, 3 µl of *Bacillus* cells (Y6 or mutants) (OD_{600} ~0.4) grown in LB medium was spotted on the PDA plate 2.5 cm away from the center, where the mycelium block was placed. Strains tested were WT (Y6, A), *ituA* (B), *fenC* (C) and *ituA fenC* (D). The antifungal activity was evaluated after 7 days of incubation at 30ºC. The inhibition diameter (the distance between Foc mycelium and bacteria colony) of WT or *fenC* mutant against Foc was measured (WT, 4.5 ± 0.5 mm; *fenC*, 2.8 ± 0.3 mm). Only minor inhibition was observed for *ituA* and *ituA fenC* mutants.

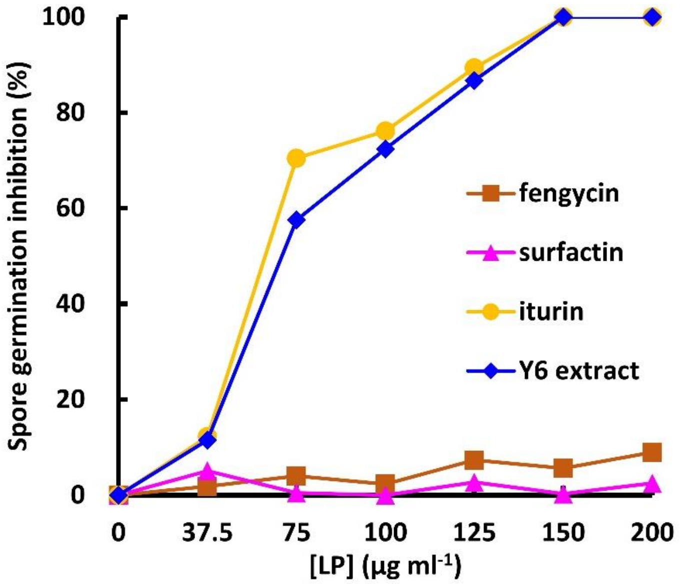

Fig. S6. Inhibitory activity of LP standards and Y6 extract against Foc spores.

A spore germination assay was conducted to test the inhibitory activity of LP standards and Y6 extract against Foc spores. Strains were inoculated in Czapek–Dox medium for two days at 30°C with continuous shaking. Cells were harvested by centrifugation and resuspended in fresh PDA medium with a final concentration of 1×10^{6} cfu ml^{-1}. One hundred microliter of the suspension was mixed with various concentration of the LP standards or Y6 extract and incubated for 12 h at 30°C. The extent of spore germination was evaluated based on five randomly selected views under a light microscope. Experiments were performed in triplicate. Data are expressed as the average percentage of spores germinated in the treated group compared to the untreated control.

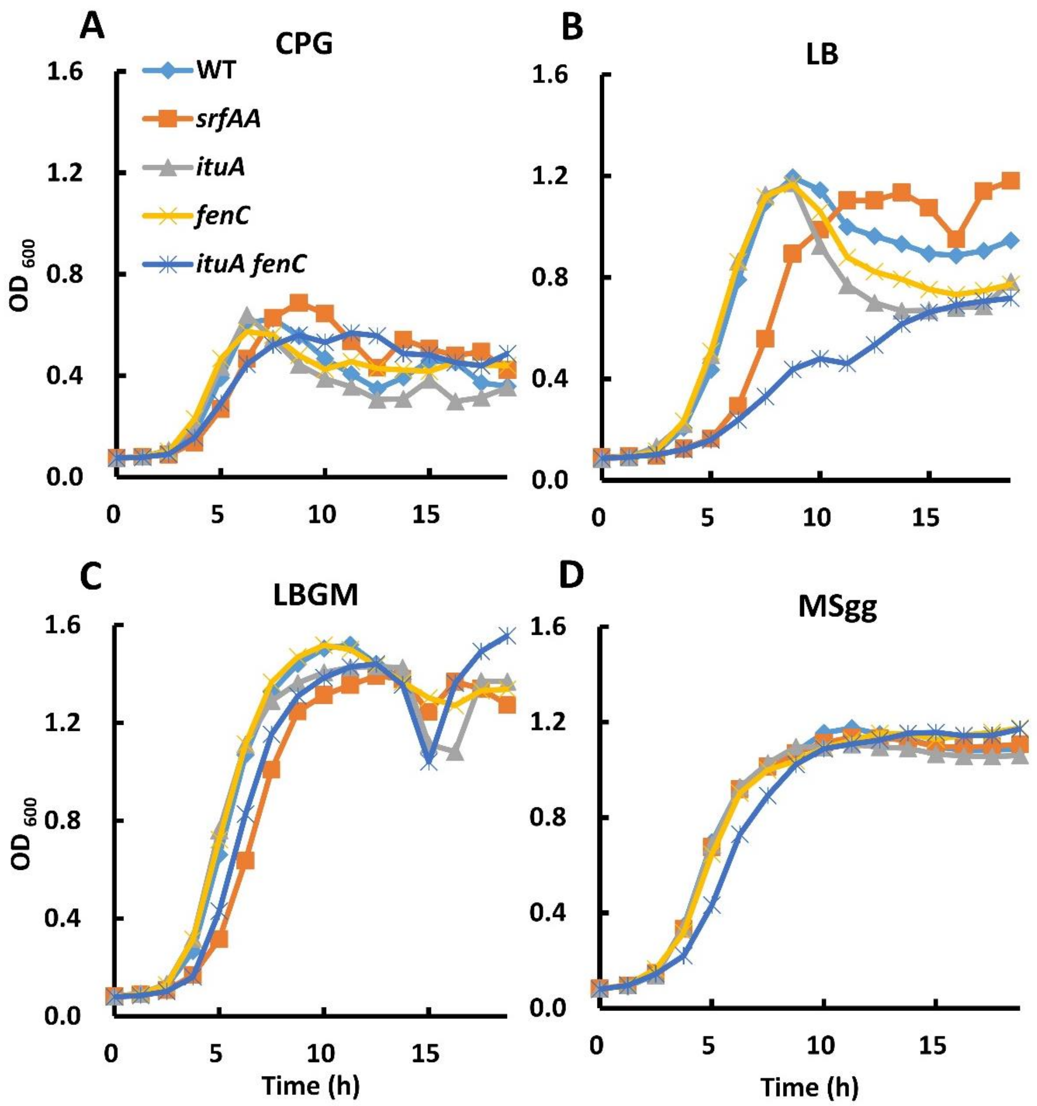

Fig. S7. Cell growth of Y6 and its derived mutants in various media.

To monitor growth, cells were grown to an OD_{600} of 0.4 in LB media. Two microliter aliquots were inoculated to 200 µl of different media as indicated in a Bioscreen 100-well microtiter plate. The media used include CPG (A), LB (B), LBGM (C) and MSgg (D). Cell growth was monitored spectrophotometrically (OD_{600}) every 15 min for 18 h using a Bioscreen growth analyzer at 30°C with continuous shaking. Data shown were representative growth curves and experiments were performed three times with three biological replicates each time.
